# Multi-View RGB Visual Sensing and Motion State Quantification of Slope Scene Visual Cues for Mobile Emergency Excavation: A Laboratory-Scale Study

**DOI:** 10.3390/s26206421

**Published:** 2026-10-11

**Authors:** Hong Weng, Yuanlong Yu

**Affiliations:** College of Computer and Data Science, Fuzhou University, Fuzhou 350108, China

**Keywords:** mobile emergency excavation, multi-view RGB visual sensing, slope scene visual cues, motion state quantification, instance segmentation, 3D reconstruction

## Abstract

Mobile emergency excavation motivates close-range sensing near unstable slopes. We evaluated a laboratory-scale red/green/blue (RGB) framework combining optimized YOLOv8n-Seg, ByteTrack and the Visual Geometry Grounded Transformer for instance perception, motion quantification and scene reconstruction. Synchronized frames and pixel-to-point correspondence linked instances to reconstructed geometry. Structural scaling and masked feature distillation yielded 1.03 million parameters and 2.04 billion floating-point operations. On the development validation subset, the mask mean average precision at intersection-over-union thresholds of 0.50–0.95 was 54.62%. Raspberry Pi 4B inference using Open Neural Network Exchange (ONNX) required 258.70 ms/image. Independent evaluation covered nine conditions with three separately prepared recordings each. Pooled Higher Order Tracking Accuracy and Identification F1 Score were 89.07 ± 0.17% and 92.50 ± 0.27% (mean ± sample standard deviation). The condition-wise mean velocity and slope root mean square errors ranged from 0.0092 to 0.0546 m/s and 2.0528° to 3.5596°, respectively. Against physical references, the mean relative dimensional errors ranged from 7.092% to 7.632%. Highlighted 3D instances, velocity heatmaps with motion vectors and occupancy density heatmaps supported scene interpretation. The results establish laboratory feasibility; moving-platform and field reliability require further validation.

## 1. Introduction

Mobile emergency excavation machinery is increasingly expected to perform road reopening, debris removal and rescue support in hazardous and spatially constrained environments. Excavators and related autonomous construction and mining platforms increasingly incorporate onboard sensing and perception to improve environmental awareness and operational safety [1,2,3,4]. However, multi-section-arm emergency excavators operating beside unstable slopes remain strongly dependent on operator vision, while long working reaches and changing boom orientations create persistent blind areas around the working face [5,6,7,8]. During emergency excavation, the platform position, viewing direction and local exposure continuously change as the machine operates. This moving observation geometry creates a close-range visual perception problem that differs from perception at fixed construction sites: usable observations must be maintained under viewpoint variation, vibration and partial occlusion as the platform moves.

Unstable slopes present a particularly difficult operating environment because relevant visual changes can be small, spatially dispersed and temporally evolving. Landslide evolution can involve progressive deformation before global instability, accompanied by observable phenomena such as increasing rockfall and vegetation disturbance [9,10,11,12,13]. These cues are context dependent and may also be influenced by machinery vibration or local excavation. Conventional inclinometers, Global Navigation Satellite System (GNSS), radar and other fixed sensing systems provide important deformation evidence [14,15,16,17,18], but their observation geometry does not follow a moving excavation platform. Video-based geohazard monitoring offers a complementary non-contact means of continuously observing visible scene changes [19]. For mobile emergency excavation, the long-term objective is therefore to develop complementary close-range visual sensing for a moving excavation platform that can characterize task-relevant scene entities and local slope geometry around the active working face, rather than to replace geotechnical monitoring. In the controlled experiments of this study, Stone and Tree instances are used as task-relevant slope scene visual entities representing rock and vegetation categories. Their movement, disturbance and redistribution are quantified as observable scene changes rather than as validated landslide precursors or calibrated landslide warning indicators.

Recent visual perception methods address complex scenes through instance segmentation, small-object enhancement and lightweight network design. Optimized YOLOv8-based segmentation improves boundary and small-object representation in cluttered agricultural scenes [20], while lightweight variants address small-object detection under resource constraints [21,22]. YOLOv8-based segmentation also supports subsequent dynamic measurement [23]. For mobile slope observation, small irregular Stone instances, occlusion and vibration-related image degradation challenge recognition accuracy and onboard computing efficiency. Motion blur and weak appearance cues further impair detection and localization [24,25]. Reliable motion quantification therefore requires a lightweight segmentation model that remains stable under these disturbances.

Single-frame instance perception, however, cannot describe how observed entities evolve. Multi-object tracking extends detections into temporally consistent identities and trajectories; ByteTrack provides an efficient association mechanism by retaining both high- and low-score detections [26], while established benchmarks show that occlusion and fast motion remain major sources of identity fragmentation [27,28]. Recent multi-object tracking research has likewise focused on maintaining robust identities in dense or occluded environments [29], and MOT-assisted multi-view reconstruction has shown that temporal association can serve as a bridge between image-space observations and object-level 3D representation [30]. For emergency excavation, this role is especially important: tracking should convert frame-wise Stone and Tree masks into continuous position, displacement, trajectory and instantaneous velocity descriptors. Temporal tracking therefore provides quantitative motion information that captures how scene entities move, rather than serving only as an ID preservation step.

Temporal descriptors alone still lack spatial context. Robotic and construction machinery perception studies increasingly combine semantic instance information with three-dimensional geometry so that detected objects can be interpreted in the scene coordinate space [4,31]. Related mobile inspection research has further shown that explicit sensor registration can connect image-space perception with metric 3D localization; Hu et al. fused LiDAR and camera data through extrinsic calibration to localize structural cracks on a mobile robotic platform [32]. Multi-view visual sensing also supports dynamic 3D measurement by exploiting synchronized observations and temporal information [33]. For structural monitoring under vibration, calibrated multi-camera systems have also emphasized explicit point cloud correction and quantitative geometric error reporting; Tang et al. validated reconstructed dimensions against laser rangefinder data [34], while Chen et al. reported millimeter-scale errors after adaptive point cloud correction [35]. Slope model studies demonstrate the value of three-dimensional reconstruction for characterizing surface geometry and deformation in controlled slope experiments [36]. At the reconstruction level, learned multi-view stereo and depth estimation methods have improved recovery of scene structure from images [37,38,39]. The Visual Geometry Grounded Transformer (VGGT) further provides a unified multi-view framework for estimating camera parameters, dense depth and consistent three-dimensional geometry from red/green/blue (RGB) images [40]. These advances establish the technical basis for image-based 3D scene reconstruction, but most existing studies emphasize either object tracking, object-level 3D perception or slope scene reconstruction as separate tasks.

Accordingly, three connected gaps remain for mobile near-field slope perception. First, object-level temporal quantification from a sensing platform whose viewpoint changes with excavation activity remains less explored in video-based geohazard observation [19,29,30]. Second, 3D perception and reconstruction methods can recover object or slope geometry [31,32,33,34,35,36,37,38,39,40], but dynamic instance motion is usually not interpreted jointly with local slope geometry. Third, lightweight small-object perception, temporal tracking and multi-view geometric reconstruction are commonly optimized as separate functions [20,21,22,23,26,29,30,31,32,33,34,35,36,37,38,39,40]. Consequently, existing RGB-based perception frameworks do not readily provide, within one processing framework, the combined information needed to determine what task-relevant entities are present, how they are moving and where that motion occurs relative to the slope surface.

As a laboratory foundation for addressing these gaps, this study develops a multi-view RGB sensing framework for semantic, temporal and geometric scene measurement with motion state quantification, intended for future integration with mobile emergency excavation machinery operating near unstable slopes. Synchronized multi-view RGB streams are used as the sole perception input and are processed through two complementary components: an instance perception and temporal tracking component for Stone and Tree instance segmentation and motion state quantification, and a slope geometry estimation and 3D reconstruction component. Their outputs are spatially aligned at the output level to generate 3D point clouds with highlighted Stone and Tree instances, velocity heatmaps with motion vectors and occupancy density heatmaps. The framework is evaluated under nine controlled laboratory-scale conditions combining three slope angles, vibration amplitudes and spatial distribution patterns. The main contributions are as follows:

(1) A lightweight instance segmentation and temporal tracking pipeline is developed for dynamic slope scenes. Static structural scaling reduces YOLOv8n-Seg complexity, MGD-inspired masked feature distillation compensates for the associated representation loss, and ByteTrack converts segmented Stone and Tree instances into cross-frame identities, trajectories and instantaneous velocities.
(2) A multi-view slope geometry estimation and 3D reconstruction component based on VGGT estimates camera parameters and depth information from synchronized RGB observations, reconstructs the observed scene and slope geometry, and provides a three-dimensional reference for interpreting instance location and motion relative to the slope surface.
(3) Output-level spatial alignment integrates instance location, motion intensity and occupancy density with reconstructed slope geometry. Evaluation across nine L9(3^3^) laboratory conditions quantifies segmentation, tracking and velocity estimation performance, together with scene- and object-level geometric accuracy.

## 2. Proposed Framework

### 2.1. Workflow

**Figure 1:**
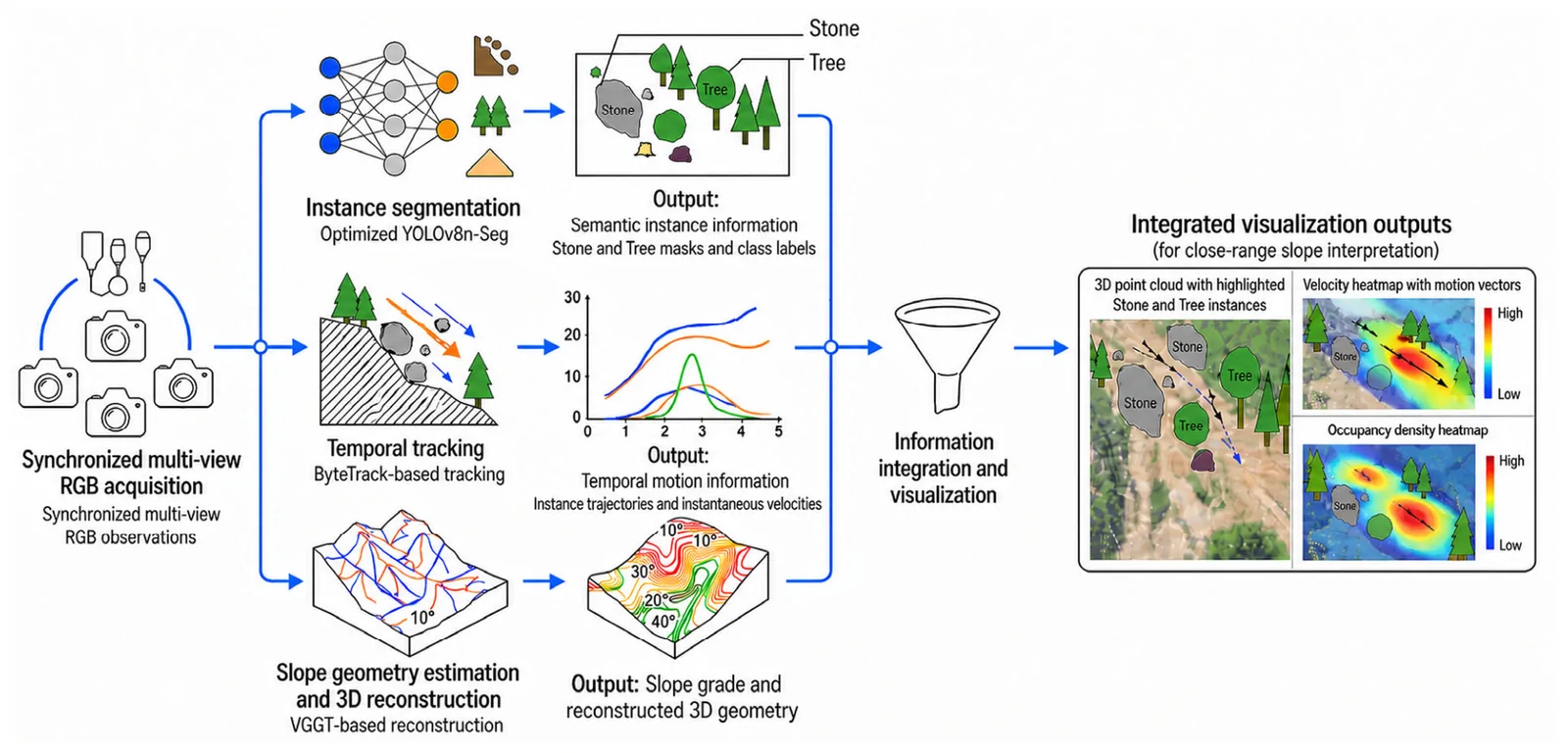
Overall workflow of the proposed multi-view RGB visual sensing framework intended for future mobile emergency excavation applications. AI-assisted explanatory artwork was created using ChatGPT (OpenAI, GPT-5.5) for schematic visualization; the technical content was verified by the authors. Blue arrows connecting the modules indicate information flow, and arrows within the scene illustrations indicate motion direction. Colored motion curves and slope geometry lines are schematic illustrations. Heatmap colors indicate relative velocity and occupancy density, as shown by the adjacent color scales.

The framework was evaluated under controlled laboratory conditions as a basis for future integration with mobile emergency excavation platforms. Synchronized multi-view RGB video streams from multiple cameras serve as the sole perception input. Figure 1 shows two parallel components for instance perception and temporal tracking, and for slope geometry estimation and 3D reconstruction. An integration module combines their outputs for local scene interpretation. This sensing–measurement–representation workflow converts RGB observations into semantic instance information, temporal motion descriptors and three-dimensional scene representations.

The first component uses optimized YOLOv8n-Seg to generate Stone and Tree masks and class labels for each RGB frame. These classes represent task-relevant visual entities, while the sand/soil mixture provides contextual slope surface information. ByteTrack associates consecutive predictions to recover trajectories and instantaneous velocities, quantifying stone movement and vegetation disturbance.

In parallel, the VGGT-based component estimates the slope angle and reconstructs the three-dimensional geometry of the observed scene from the same synchronized multi-view RGB observations. The reconstructed slope geometry provides the spatial context required to interpret the locations and motions of Stone and Tree instances relative to the slope surface.

At the information integration and visualization stage, Stone and Tree instance position and velocity information are combined with reconstructed 3D geometry and slope information to generate 3D point clouds with highlighted Stone and Tree instances, velocity heatmaps with motion vectors and occupancy density heatmaps. By showing where Stone and Tree instances are concentrated and when local motion responses intensify, these integrated visualization outputs provide spatially and temporally resolved information for operator-oriented scene interpretation.

### 2.2. Core MODULES

#### 2.2.1. Instance Perception and Temporal Tracking Module

**Figure 2:**
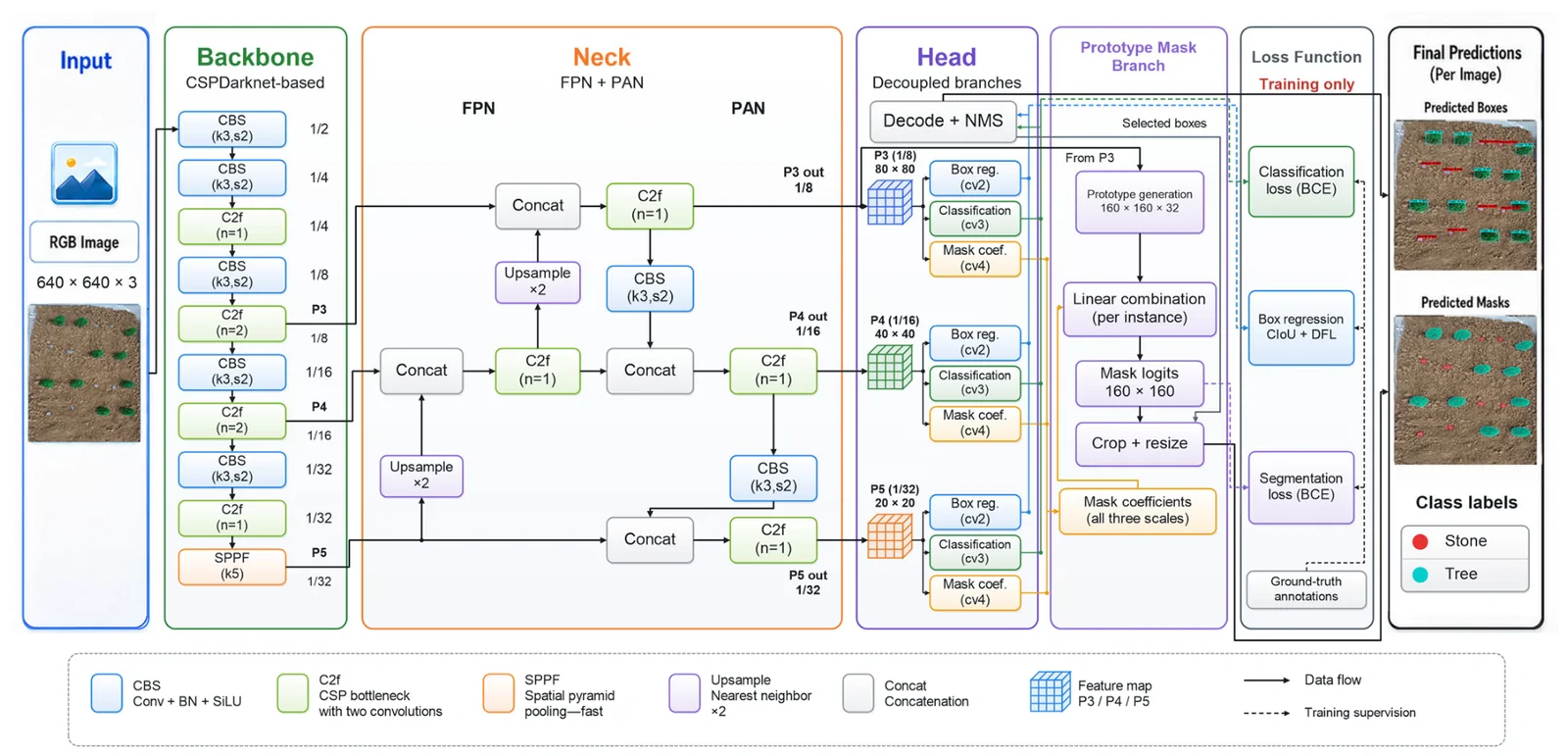
Basic architecture of the YOLOv8n-Seg baseline instance segmentation network. AI-assisted explanatory artwork was created using ChatGPT (OpenAI, GPT-5.5) for schematic visualization; the network architecture and data flow were verified by the authors.

YOLOv8n-Seg provides the baseline architecture shown in Figure 2, with normalized RGB inputs resized to 640 × 640 × 3. The backbone uses CBS (convolution-batch normalization-SiLU), C2f (cross-stage partial bottleneck with two convolutions) and SPPF (spatial pyramid pooling—fast) components. It produces feature maps at 1/8, 1/16 and 1/32 scales. A feature pyramid network–path aggregation network (FPN–PAN) neck fuses P3–P5 features to represent Stone and Tree instances of different sizes. The decoupled head predicts classes, bounding boxes and mask coefficients, which combine with the prototype mask branch to produce instance masks. Training uses classification, bounding-box regression, distribution focal and mask losses. Static structural scaling (SSS) reduces complexity, and a feature distillation strategy inspired by Masked Generative Distillation (MGD) compensates for representation loss [41].

Static structural scaling reduced YOLOv8n-Seg complexity by changing the width multiplier from 0.25 to *w* = 0.125 while retaining the depth multiplier *d* = 0.33 [42]. In Equations (1) and (2), $C_{\mathrm{base}}$ and $R_{\mathrm{base}}$ denote channel counts and layer repeats before applying these multipliers. The resulting architecture served as the lightweight student for feature distillation. For the trained-baseline variants, compatible weights were loaded from the trained YOLOv8n-Seg baseline checkpoint; other parameters retained their initial values.

(1) $$C_{\mathrm{scaled}}=\max\left(8,\mathrm{round}\left(C_{\mathrm{base}}\;\times \;w\right)\right)$$

(2) $$R_{\mathrm{scaled}}=\max\left(1,\mathrm{round}\left(R_{\mathrm{base}}\;\times \;d\right)\right)$$

To compensate for scaling-related representation loss, the unscaled YOLOv8n-Seg teacher supervises the lightweight student through MGD-inspired masked feature distillation [41,43,44]. Supervision uses six outputs from the cv2 box regression and cv3 classification branches at 80 × 80, 40 × 40 and 20 × 20 pixels. A random spatial binary mask sets approximately 65% of student positions to zero (masking ratio 0.65) and retains 35%. The masked student output is aligned directly with its paired teacher output by mean squared error, without the generator used in canonical MGD. This supervision adds no inference cost because the teacher and masking operate only during training. In Equations (3) and (4), *K* counts valid output pairs. $F_{k}^{S}$ and $F_{k}^{T}$ denote student and teacher features at level *k*, respectively. The binary mask $M_{k}$ applies through element-wise multiplication (⊙) to the corresponding student output.

(3) $$L_{\mathrm{total}}=L_{\mathrm{task}}+\beta L_{\mathrm{mask}}$$

(4) $$L_{\mathrm{mask}}=\frac{\alpha }{K}\sum_{k=1}^{K}\mathrm{MSE}(F_{k}^{S}⊙M_{k},F_{k}^{T})$$

In Equation (4), MSE averages over all *B* × *C* × *H* × *W* elements, including masked positions; losses are then averaged over *K* valid pairs. The training criterion multiplies the weighted task loss by batch size, while the distillation term has no additional batch size multiplier. The internal feature loss weight *α* = 0.1 and outer coefficient *β* = 0.1 yield an effective feature error coefficient of 0.01. Table 1 records the implementation, normalization and training settings for reproducibility.

ByteTrack associates the bounding boxes and confidence scores accompanying the optimized YOLOv8n-Seg predictions across frames [26]. A Kalman filter predicts motion states [45], and intersection over union (IoU) and the Hungarian algorithm support instance association [46]. Association uses motion predictions and geometric overlap without appearance-based re-identification. The instance masks retain the associated track IDs. Outputs include trajectories, positions, displacements, velocities and trajectory continuity, which are linked to VGGT scene geometry as described in Section 2.2.3.

#### 2.2.2. Slope Geometry Estimation and 3D Reconstruction Module

**Figure 3:**
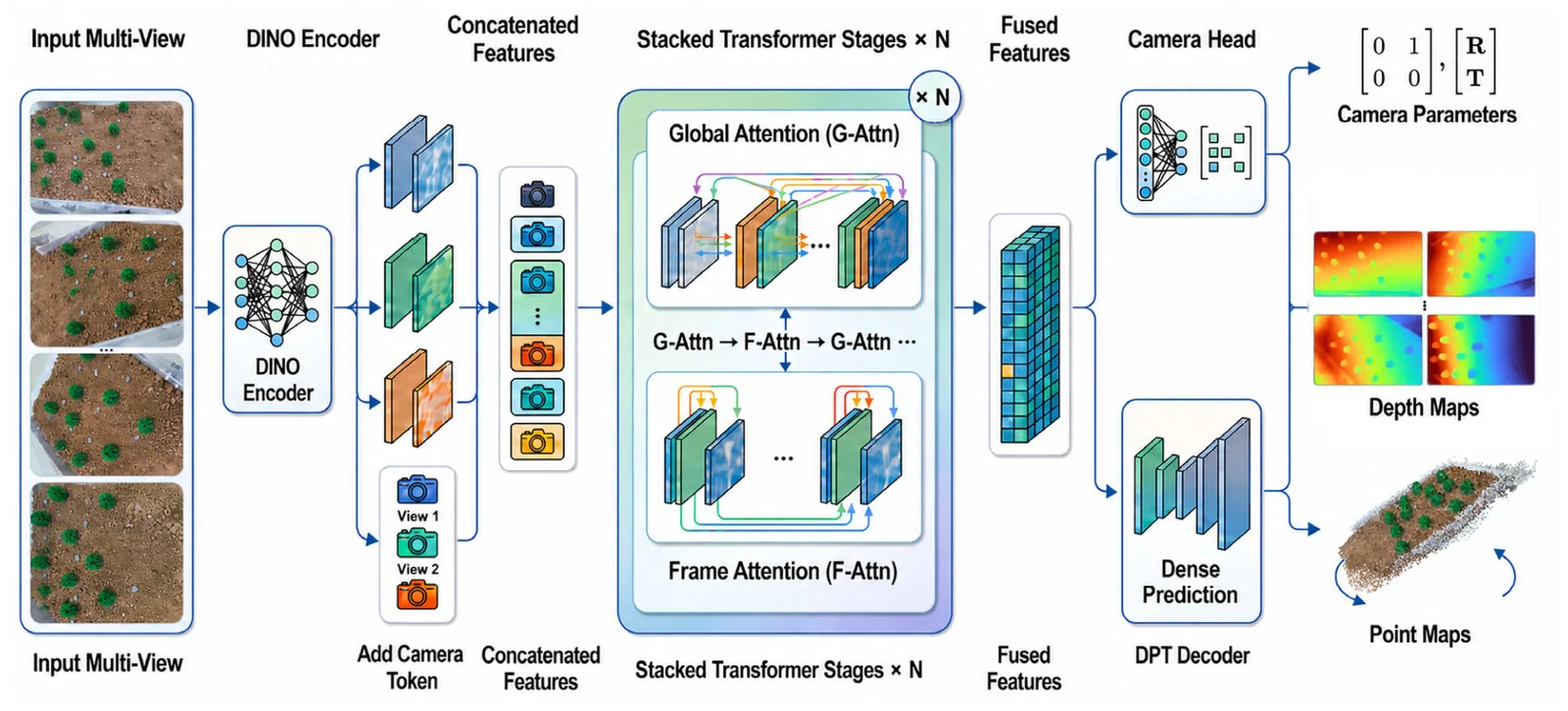
Overview of the VGGT architecture used for slope geometry estimation and 3D reconstruction, based on Wang et al. [40]. AI-assisted explanatory artwork was created using ChatGPT (OpenAI, GPT-5.5) for schematic visualization; the architectural components and data flow were verified by the authors. Arrows indicate feature processing and information flow; colors distinguish view-specific feature representations. ×N denotes repeated transformer stages. Four input images and corresponding depth maps are shown for illustration; ellipses in the input sequence and between the depth map rows indicate additional views not shown.

The slope geometry estimation and 3D reconstruction module employs VGGT to estimate camera parameters, depth information and three-dimensional scene geometry from synchronized multi-view RGB images. Each input image is patchified and encoded into image tokens through a DINO-based backbone. The tokens are subsequently processed by a Transformer that alternates between frame-wise and global self-attention, enabling both within-view feature refinement and cross-view geometric reasoning. The overall VGGT architecture is shown in Figure 3 [40].

Learnable camera tokens, which implicitly encode view-specific camera information, are appended to the corresponding image tokens before they are jointly passed to the geometric reasoning network. This token representation enables the model to preserve view-specific visual information while establishing a common feature space for subsequent cross-view reasoning.

Within this geometric reasoning process, frame attention and global attention are applied alternately across the 24-layer core network. Frame attention performs self-attention independently within the token set of each image, where the corresponding camera token and image tokens form a local sequence. This operation preserves single-view spatial structure and local details before cross-view fusion. Global attention then concatenates the token sequences from all views into a unified global sequence and performs cross-view self-attention, thereby establishing cross-view feature associations and capturing geometric consistency across views. The outputs of the two attention operations are fused through concatenation, linear projection and residual addition, allowing the representation to evolve progressively from view-specific features to a globally consistent geometric description. This global context supports subsequent camera pose estimation and three-dimensional reconstruction. The attention operation is expressed as Equation (5):

(5) $$\mathrm{Attention}\left(Q,K,V\right)\;=\;\mathrm{softmax}\left(\frac{QK^{T}}{\sqrt{d_{k}}}\right)V$$

In Equation (5), *Q*, *K* and *V* are generated from the local token sequences of individual images for frame attention and from the concatenated multi-view sequence for global attention, while $d_{k}$ denotes the dimensionality of the key vectors.

After the attention stages, the camera head decodes camera tokens to estimate the intrinsic and extrinsic parameters of each view. The dense prediction transformer (DPT) decoder transforms image tokens into dense feature maps for depth prediction. The estimated camera parameters and depth maps are then combined to reconstruct the scene point cloud. After excluding Stone and Tree points and obvious outliers, RANSAC plane fitting estimates the normal of the selected slope surface. The horizontal reference normal is estimated from reconstructed points on a fixed surface, confirmed to be horizontal and visible in the same input images. Both normals are expressed in the same reconstructed coordinate system, and the predicted slope angle is calculated as the acute angle between them.

#### 2.2.3. Information Integration and Visualization Module

This module combines Stone and Tree instance segmentation and tracking information, including category, position, trajectory and velocity, with the slope geometry and 3D reconstruction results provided by VGGT. The key link between the two processing branches is the pixel-to-point correspondence established from the same synchronized RGB frames. For each input frame, VGGT estimates the camera parameters and depth information, reconstructs the corresponding three-dimensional scene geometry, and retains the correspondence between each valid image pixel and its reconstructed three-dimensional point. In parallel, the optimized YOLOv8n-Seg model (SSS + MGD) processes the same RGB frame to obtain Stone and Tree instance masks. Because the segmentation model operates on resized inputs, each predicted mask is first restored to the pixel grid of the corresponding VGGT input image. The three-dimensional points associated with pixels inside each restored mask are then assigned the corresponding instance label and highlighted in the reconstructed point cloud. In this way, VGGT provides the geometric reference that links two-dimensional instance perception to the reconstructed three-dimensional scene without requiring separate target-based camera calibration or post hoc registration between the two processing branches. ByteTrack track identities and motion attributes are subsequently associated with the corresponding highlighted instances, allowing their trajectories and velocities to be interpreted relative to the reconstructed slope surface.

The spatially aligned outputs are displayed as 3D point clouds with highlighted Stone and Tree instances, velocity heatmaps with motion vectors and occupancy density heatmaps. Velocity heatmaps encode relative motion intensity, with arrow direction and length indicating motion trend and magnitude. Occupancy density heatmaps represent the relative aggregation of Stone and Tree instances. Together, these products show instance location, motion intensity and spatial concentration for local scene interpretation under the tested laboratory conditions.

## 3. Experiments

### 3.1. Experimental Platform and Working Condition Design

A rectangular slope model apparatus with an adjustable angle was used to reproduce selected visible movements under controlled laboratory conditions. These included slow tilting, rolling stones, tree disturbance and localized accelerated sliding of the sand/soil surface. The artificial entities and controlled motions were used to evaluate visual sensing and were not intended as validated physical proxies for landslide precursors. The experiment evaluated instance segmentation, temporal tracking, motion state quantification, slope geometry estimation and 3D reconstruction, and integrated visualization across the prescribed working conditions.

The laboratory setup was stationary and did not include an excavator-mounted moving sensing platform. The imposed vibration conditions were designed to introduce controlled visual disturbances within the laboratory setup.

**Figure 4:**
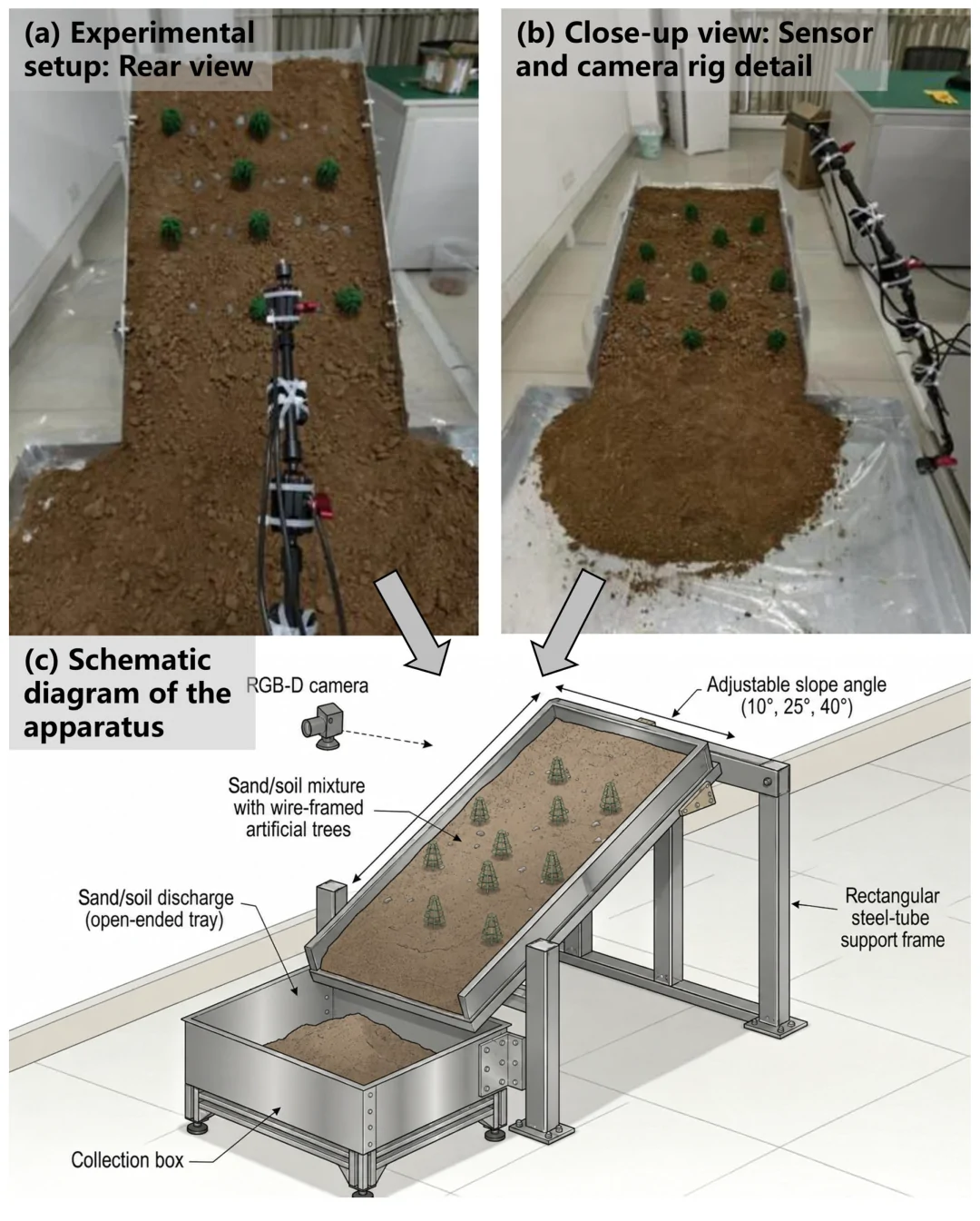
Experimental apparatus: (**a**) rear view of the experimental setup; (**b**) close-up view of the sensor and camera rig; (**c**) schematic illustration of the apparatus. Panels (**a**,**b**) are original experimental photographs and were not generated or altered using generative AI. Panel (**c**) was created with AI assistance using ChatGPT (OpenAI, GPT-5.5) for schematic visualization; the apparatus configuration was verified by the authors.

The experimental apparatus adopts a hinged structure consisting of a fixed base frame and a movable slope surface. The slope angle is adjusted using angle-limiting plates and set to three levels: 10°, 25° and 40°. The frame is composed of rectangular steel tubes and steel plates. Photographs and a schematic illustration of the apparatus are shown in Figure 4. Although RGB-D cameras were used as the acquisition hardware, only their RGB streams were used as perception inputs in the proposed framework.

The experimental materials comprised wire-framed artificial trees, Qinglong stones used as rockfall analogues and a sand/soil mixture. The trees had total heights of 10–28 cm, including their trunks, which were partially inserted into the slope during scene preparation. Three stone size categories were prepared: <2 cm particles, 2–5 cm blocks and 5–8 cm large blocks. The nine L9 working conditions consistently used the 2–5 cm block type.

The experimental design considered three factors at three levels: the spatial distribution pattern of Stone and Tree instances (multi-center clustered, checkerboard grid and random patch), slope angle (10°, 25° and 40°), and vibration amplitude (0.1 g, 0.3 g and 0.5 g). The apparatus hardware, sand/soil composition and stone size were held constant across all working conditions. Nine representative working conditions were defined using an L9(3^3^) orthogonal array, as listed in Table 2.

Each working condition was repeated in three independently prepared experimental runs. Within a working condition, the prescribed slope angle and vibration amplitude were maintained across the three runs. Before each run, the sand/soil slope surface was re-prepared, the Stone and Tree instances were rearranged according to the prescribed spatial distribution pattern, and image acquisition was restarted. For the L9 condition-wise evaluation, 27 experimental runs were conducted, with the run rather than the individual image serving as the unit of experimental replication (*n* = 3 per working condition).

**Figure 5:**
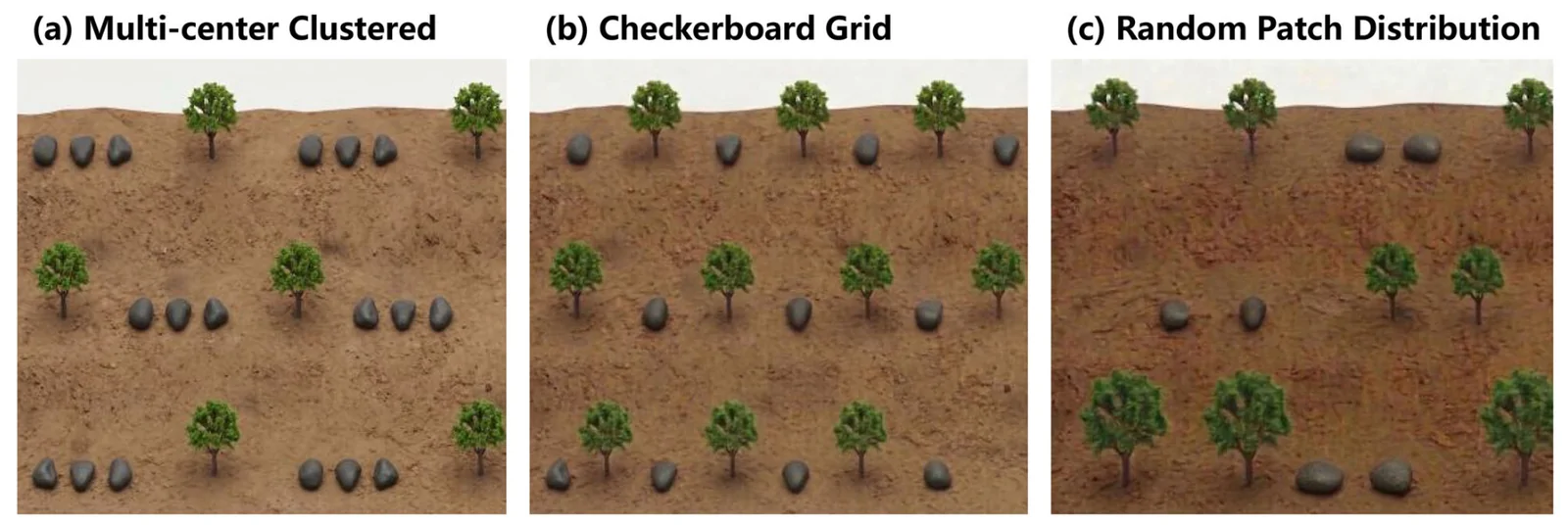
Schematic illustrations of the spatial distribution patterns used in the experimental design: (**a**) multi-center clustered distribution; (**b**) checkerboard grid distribution; (**c**) random patch distribution. AI-assisted explanatory artwork was created using ChatGPT (OpenAI, GPT-5.5) to visualize the prescribed spatial arrangements; the distribution logic was verified by the authors.

The three representative spatial distribution patterns used in the experimental design are schematically illustrated in Figure 5.

### 3.2. Dataset Construction

Two independently collected RGB datasets were used for YOLOv8n-Seg development and system evaluation, respectively. The development dataset comprised 27 video sequences, divided into 21 training, 3 validation and 3 test sequences (7:1:1). These subsets contained 4031, 568 and 602 images, respectively, totaling 5201 images. All images extracted from a given video were assigned to the same subset. Motion blur and brightness augmentation were applied only to training images after partitioning. The independent evaluation dataset comprised three separately acquired recordings per L9 working condition (C1–C9), totaling 27 experimental runs. These recordings were excluded from model development and used to evaluate segmentation, tracking, motion state quantification and geometric reconstruction.

Preprocessing included denoising, frame alignment and temporal synchronization. One annotator manually labeled Stone and Tree instance masks in LabelMe, reviewed every frame and corrected detected errors. The sand/soil mixture provided contextual slope surface information. Ground-truth track IDs were assigned manually by identifying the same physical objects across frames. Independent velocity ground truth was obtained by manually tracking instance centers. Manually tracked image centers ($u_{t}$, $v_{t}$) were mapped to metric slope plane positions ($X_{t}$, $Y_{t}$) by a planar homography. Four fixed reference points with known metric coordinates defined this homography. Ground-truth velocities were calculated from consecutive metric displacements at 30 fps (1/30 s per frame). The same center-point, coordinate and sampling definitions were used for predicted trajectories. Velocity RMSE used only continuous, identity-consistent matched trajectory samples. No interpolation was performed across missed detections, identity switches or track gaps. Calculation resumed only after a continuous, identity-consistent track segment was re-established. Tracking and motion state metrics were summarized as the mean ± sample standard deviation (SD) across the three recordings per condition. Slope angle ground truth was obtained independently with an inclinometer.

Each VGGT reconstruction was aligned to the 1.00 m × 0.57 m rectangular slope using four manually selected corners in each run. Their reference coordinates were *P*1 = (0, 0, 0), *P*2 = (0.57, 0, 0), *P*3 = (0.57, 1.00, 0) and *P*4 = (0, 1.00, 0). The *X* axis runs from left to right, the *Y* axis from the slope toe to crest, and the *Z* axis is normal to the slope. Singular value decomposition (SVD) was used to fit the three-dimensional similarity transformation, $X_{\mathrm{metric}}=sRX_{\mathrm{VGGT}}+t$. This transformation recovered a uniform metric scale, rotation and translation for each run, establishing the slope coordinate system for object dimensional and slope plane positional measurements. Optimized YOLOv8n-Seg (SSS + MGD) masks from the same RGB frames were restored to the VGGT pixel grid. Instance point clouds were then extracted through the retained pixel-to-point correspondence.

Principal component analysis (PCA) defined the main and orthogonal axes of each instance point cloud projected onto the slope XY plane. Stone main-axis length and orthogonal width, and Tree main and orthogonal crown widths, were estimated from the 1st–99th percentile spans along these axes. Vernier-caliper ground truth comprised the maximum projected Stone length and Tree crown width and their perpendicular spans in the slope plane. Tree crown endpoints were manually marked and projected normally onto this plane. Crown height was the slope-normal distance between the lower and upper crown boundaries. It was measured with a vernier caliper between the marked endpoints and predicted from the maximum minus minimum Z coordinate of the reconstructed crown points. For position validation, the projected Stone geometric center and Tree base were measured as caliper distances from the left and lower slope boundaries. The local slope reference was the median Z of at least 20 valid non-instance points in a 12-pixel ring around each Tree mask, excluding all Stone and Tree masks. Predicted Stone positions were robust XY centers of the instance point clouds. Predicted Tree base positions were XY medians of the 8% of Tree points closest to the local slope reference. Points with non-finite coordinates were removed, and instances with fewer than 20 valid points were excluded from geometric measurement.

Training used Windows 11, an AMD Ryzen 9 9950X 16-Core Processor and a single NVIDIA GeForce RTX 5060 Ti GPU. Software comprised CUDA 12.8, Python 3.10.4 and PyTorch 2.10.0.

Generative AI (ChatGPT, OpenAI, GPT-5.5) assisted language refinement and explanatory artwork in Figure 1, Figure 2, Figure 3, Figure 4c and Figure 5. Experimental data and quantitative results were generated independently of generative AI; the full usage and author verification statement is provided in the Acknowledgments.

## 4. Results and Discussion

### 4.1. Evaluation Metrics

To evaluate the proposed framework, quantitative metrics were defined for Stone and Tree instance segmentation, temporal tracking, motion state quantification and slope geometry estimation and 3D reconstruction. The integrated visualization outputs are assessed qualitatively and interpreted in conjunction with the quantitatively evaluated module-level results. The L9 array defined nine working conditions. Quantitative analyses used the independent experimental runs to characterize variability within each working condition. Factor-level summaries from the L9 array were treated descriptively and were not used to infer independent main or interaction effects.

For the YOLOv8n-Seg instance segmentation module, mean average precision (mAP) was the core evaluation metric [47]. Average precision (AP) summarizes the precision–recall curve for each class. mAP50 uses IoU = 0.50; mAP50–95 averages mAP over IoU thresholds from 0.50 to 0.95 in steps of 0.05. Equation (6) defines mAP at each IoU threshold as the arithmetic mean of class-specific AP values, with *N* = 2 for Stone and Tree:

(6) $$\mathrm{mAP}=\frac{1}{N}\sum_{i=1}^{N}AP_{i}$$

In addition, IoU is used to quantify mask overlap [48], while precision, recall and F1 score are reported as auxiliary metrics. Together, these metrics provide a multidimensional characterization of segmentation boundary accuracy and category classification capability, as shown in Equations (7)–(10):

(7) $$\mathrm{IoU}\;=\frac{\mathrm{Area}\left(A\cap B\right)}{\mathrm{Area}\left(A\cup B\right)}$$

(8) $$P=\frac{\mathrm{TP}}{\mathrm{TP}+\mathrm{FP}}$$

(9) $$R=\frac{\mathrm{TP}}{\mathrm{TP}+\mathrm{FN}}$$

(10) $$F_{1}=\frac{2\mathrm{PR}}{P+R}$$

For the ByteTrack-based tracking submodule, standard multi-object tracking metrics were calculated with TrackEval from human-reviewed frame-level ground-truth trajectories and corresponding predictions. Stone and Tree were evaluated separately using two-dimensional bounding-box IoU for frame-level matching. Metrics included Higher Order Tracking Accuracy (HOTA), Association Precision (AssPr) and Association Recall (AssRe). Identification F1 Score (IDF1), identity switches (IDSW) and trajectory fragmentations (Frag) were also evaluated; IDSW and Frag were reported as normalized event rates. HOTA, AssPr and AssRe were averaged over IoU thresholds from 0.05 to 0.95 in steps of 0.05. At each threshold, detection accuracy (DetA), association accuracy (AssA) and HOTA were calculated as

(11) $$\begin{array}{c} \mathrm{DetA}=\frac{\left|TP\right|}{\left|TP|+|FP|+|FN\right|},\;\mathrm{AssA}=\frac{1}{\left|TP\right|}\sum_{c\in TP}\frac{TPA(c)}{TPA(c)+FPA(c)+FNA(c)}\\ \mathrm{HOTA}=\sqrt{DetA\cdot AssA} \end{array}$$

while AssPr and AssRe were calculated as

(12) $$\begin{array}{c} \mathrm{AssPr}=\frac{1}{\left|TP\right|}\sum_{c\in TP}\frac{TPA(c)}{TPA(c)+FPA(c)}\\ \mathrm{AssRe}=\frac{1}{\left|TP\right|}\sum_{c\in TP}\frac{TPA(c)}{TPA(c)+FNA(c)} \end{array}$$

Here, TP, FP and FN are sets of matched, unmatched predicted and unmatched ground-truth detections, respectively; vertical bars denote set size. For each match *c*, TPA(*c*), FPA(*c*) and FNA(*c*) count correct, extra and missed associations for its ground-truth/predicted trajectory pair [49]. AssA, AssPr and AssRe average the corresponding trajectory pair ratios over all matched detections. IDF1 was evaluated at IoU = 0.5 as

(13) $$IDF1=\frac{2IDTP}{2IDTP+IDFP+IDFN}$$

where IDTP, IDFP and IDFN denote identity true positives, identity false positives and identity false negatives, respectively [50]. At the same IoU threshold, an IDSW event was counted when a ground-truth target was matched to a different predicted track ID from its previous assignment, whereas a Frag event was counted when a previously matched ground-truth trajectory became unmatched and was subsequently matched again [51]. The corresponding normalized event rates were calculated for each recording as

(14) $$R_{E}=\frac{N_{E}}{N_{GT}}\times 1000,E\in \left\{IDSW,Frag\right\}$$

Here, $N_{E}$ denotes the corresponding IDSW or Frag event count, and $N_{GT}$ denotes the total number of ground-truth detections across all frames in the recording. The normalized value $R_{E}$ is expressed as events per 1000 GT detections.

The standard MOT metrics defined above were calculated separately for each independent recording. Stone and Tree results were combined into the Overall result using TrackEval’s metric-specific class aggregation. For each working condition, the three run-level values were summarized as the mean ± sample SD. For the pooled C1–C9 evaluation, the nine corresponding recordings were aggregated separately for each of R1–R3 before summarizing the three replicate-level pooled results. IDSW and Frag event counts and ground-truth detections were summed across C1–C9 for each of R1–R3 before applying Equation (14).

TSR and NETR were retained as application-specific auxiliary measures alongside the standard tracking metrics. TSR is the proportion of ground-truth targets for which a single predicted track ID matches more than 80% of the target’s visible ground-truth frames. NETR measures valid predicted track IDs in excess of the physical target count; a predicted ID is considered valid if it is detected in more than five frames. TSR and NETR are defined in Equations (15) and (16):

(15) $$\mathrm{TSR}\;=\frac{N_{\mathrm{tracked}}}{N_{\mathrm{gt}}}$$

(16) $$NETR=\frac{\max\left(0,N_{valid}-N_{actual}\right)}{N_{actual}}$$

Here, $N_{tracked}$ denotes the number of successfully tracked targets, $N_{gt}$ denotes the total number of ground-truth targets, $N_{valid}$ denotes the number of predicted track IDs detected in more than five frames, and $N_{actual}$ is the number of physical targets placed in the experimental scene. A higher NETR indicates more severe excess track-ID generation, which may be associated with trajectory fragmentation, repeated track initialization or persistent false-positive tracks. NETR is therefore interpreted as an application-specific diagnostic indicator rather than as the conventional frame-level IDSW metric.

Root mean square error (RMSE) quantified instantaneous velocity estimation error for Stone and Tree instances. Estimated and ground-truth velocities were compared in the same metric slope plane coordinate system using continuous, identity-consistent matched samples.

For the VGGT-based slope geometry estimation and 3D reconstruction module, geometric accuracy was evaluated at both scene and object levels. Scene-level slope estimation was measured using mean absolute error (MAE), which is less sensitive to outliers, and root mean square error (RMSE), which is sensitive to larger deviations, as shown in Equations (17) and (18). The complete scene point clouds were additionally examined qualitatively to complement the quantitative geometric measures; broader reconstruction-assessment strategies have also been proposed for 3D mapping evaluation [52].

(17) $$\mathrm{MAE}=\frac{1}{n}\sum_{i=1}^{n}\left|\theta_{i}-{\hat{\theta }}_{i}\right|$$

(18) $$\mathrm{RMSE}=\sqrt{\frac{1}{n}\sum_{i=1}^{n}{\left(\theta_{i}-{\hat{\theta }}_{i}\right)}^{2}}$$

These metrics quantify slope estimation accuracy under the tested conditions. For each of the 27 experimental runs, VGGT estimated the slope angle from multi-view RGB inputs at each evaluable frame. The same frame inclusion criteria, preprocessing, VGGT settings and angle extraction procedure were applied throughout, using the run-specific inclinometer reading as ground truth. The RMSE and MAE were computed separately within each run. The three recordings per working condition were treated as independent experimental replicates. Their run-level RMSE and MAE values were summarized as the mean ± sample SD to quantify variation across repeated acquisitions.

Object-level geometric accuracy was evaluated from the metric-scale Stone and Tree instance point clouds using independent physical measurements. For each evaluated dimension, the MAE was calculated by averaging the absolute differences between predicted and measured values. The mean relative error (MRE) was calculated by first normalizing each absolute dimensional error by its corresponding ground-truth value and then averaging over valid instances, as shown in Equation (19). The slope plane positional RMSE was calculated from the squared XY errors in Equation (20).

(19) $$\mathrm{MRE}=\frac{1}{N}\sum_{i=1}^{N}\frac{\left|{\hat{d}}_{i}-d_{i}\right|}{d_{i}}\times 100\%$$

(20) $${\mathrm{RMSE}}_{\mathrm{pos}}=\sqrt{\frac{1}{N}\sum_{i=1}^{N}\left[{\left({\hat{x}}_{i}-x_{i}\right)}^{2}+{\left({\hat{y}}_{i}-y_{i}\right)}^{2}\right]}$$

Here, *N* is the number of valid instances of each class included in the calculation; $d_{i},{\hat{d}}_{i}$ denote measured and predicted dimensions, respectively, and $\left(x_{i},y_{i}\right),\left({\hat{x}}_{i},{\hat{y}}_{i}\right)$ denote the corresponding reference and predicted slope plane positions. For each working condition, the R1–R3 run-level metrics were summarized as the mean ± sample SD. For the pooled C1–C9 evaluation, the valid instances from the nine corresponding recordings were pooled separately for each of R1–R3; the resulting three values for each metric were summarized as the mean ± sample SD (*n* = 3).

### 4.2. Optimization Effect of the Instance Segmentation Model

Table 3 compares YOLOv8n-Seg, YOLO11n-Seg and YOLO26n-Seg with three staged YOLOv8n-Seg variants on the same model development validation subset. All six configurations used the same 150-epoch training budget. Lightweight refers to lower parameter count, computational cost measured in billions of floating-point operations (GFLOPs), and model size, considered alongside device-specific inference time. Under matched random initialization, SSS reduced parameters from 3.26 M to 1.03 M, GFLOPs from 6.06 to 2.04 and model size from 6.46 to 2.20 MB. Box and Mask mAP50–95 decreased by 10.05 and 1.59 percentage points, respectively, quantifying the accuracy cost of structural scaling.

For the same SSS architecture and 150-epoch schedule, trained-baseline initialization recovered 2.65 percentage points in Box mAP50–95 and 0.42 in Mask mAP50–95. With architecture, initialization and the 150-epoch schedule unchanged, MGD added 5.20 and 0.20 percentage points, respectively. The larger Box gain was consistent with supervision of cv2 regression and cv3 classification outputs rather than the prototype mask branch. These stages distinguish compression by SSS, recovery through initialization and further improvement through distillation.

Raspberry Pi 4B inference used the Open Neural Network Exchange (ONNX) format. SSS + MGD reduced inference time from 639.90 to 258.70 ms/image (59.6%) relative to unscaled YOLOv8n-Seg. RTX 5060 Ti inference time decreased from 4.71 to 4.28 ms/image, showing a smaller GPU speed gain. These single-image timings assess model inference; end-to-end multi-view latency and sustained throughput on a moving platform remain unevaluated.

**Figure 6:**
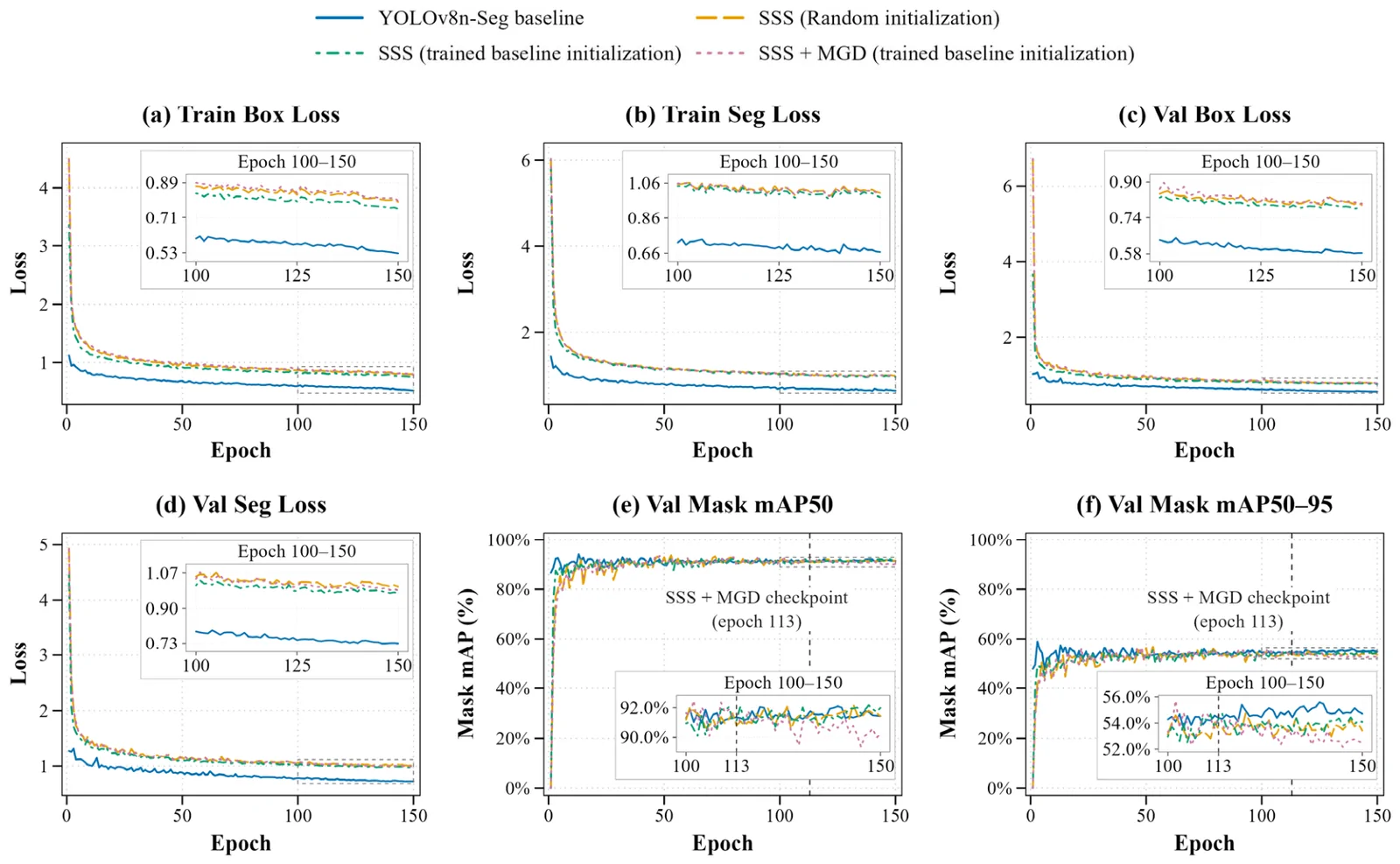
Training and validation dynamics of four YOLOv8n-Seg variants over 150 epochs: (**a**,**b**) training box and segmentation loss; (**c**,**d**) validation box and segmentation loss; (**e**,**f**) validation Mask mAP50 and mAP50–95. Insets show epochs 100–150; vertical dashed lines mark the SSS + MGD checkpoint at epoch 113.

**Figure 7:**
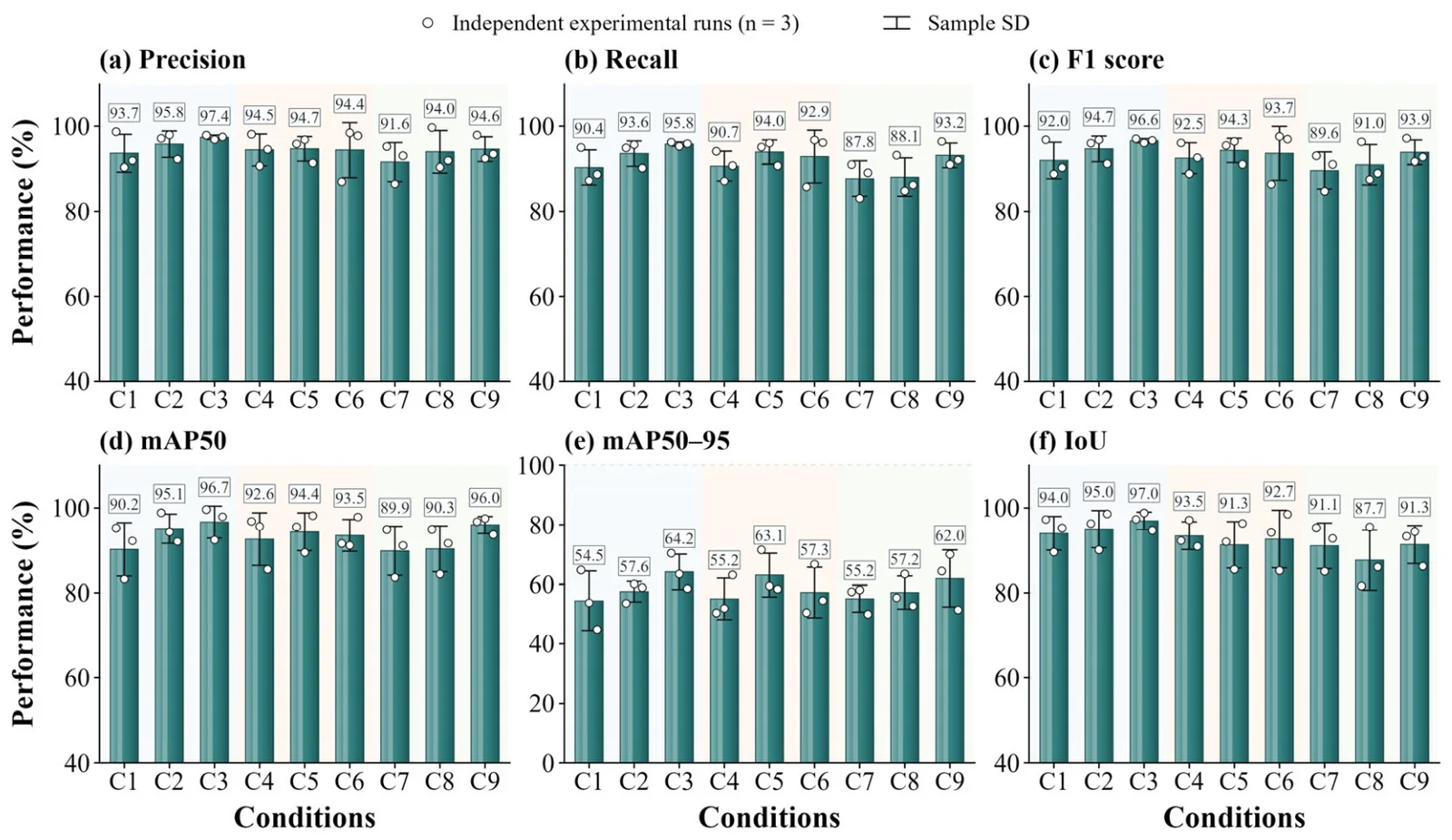
Overall instance segmentation performance under different working conditions (three independent experimental runs per condition). Background shading groups C1–C3 (light blue, 10°), C4–C6 (light orange, 25°) and C7–C9 (light green, 40°).

**Figure 8:**
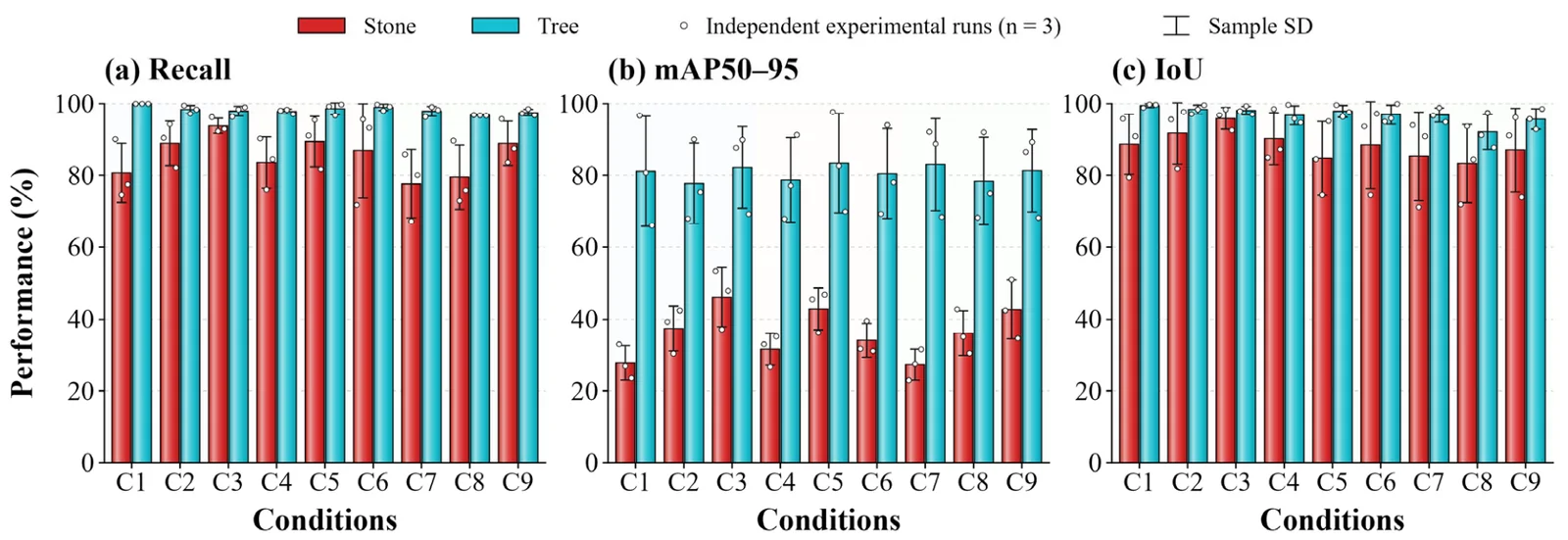
Class-level segmentation performance of Stone and Tree under different working conditions (three independent experimental runs per condition).

Figure 6 compares training losses, validation losses and validation Mask mAP for the four YOLOv8n-Seg variants over 150 epochs. Training box and segmentation losses continued to decrease, while validation losses flattened and remained higher for scaled models than for the unscaled baseline. The SSS + MGD checkpoint selected at epoch 113 achieved 54.62% validation Mask mAP50–95 (Table 3). By epoch 150, this score was approximately two percentage points lower, and Mask mAP50 also declined despite decreasing training losses. This divergence supports earlier checkpoint selection; Figure 7 and Figure 8 separately assess performance across the nine dynamic conditions.

### 4.3. Module-Wise Performance of the Framework

#### 4.3.1. Two-Dimensional Instance Segmentation Performance

Figure 7 reports optimized YOLOv8n-Seg results on the independent L9 recordings, separately from the model development validation results in Table 3. Three run-level values are shown per condition; the following averages summarize the nine condition-wise means. Precision, recall and F1 score averaged 94.52%, 91.81% and 93.15%, respectively. Precision exceeded recall by 2.71 percentage points, suggesting a greater limitation from missed detections than false positives. The mean mAP50 was 93.20% (range 89.88–96.67%), and the mean mAP50–95 was 58.45% (range 54.46–64.17%). The 34.75-percentage-point gap between mAP50 and mAP50–95, together with variation among runs, indicates a limitation in precise mask localization. The mean IoU across the nine condition-wise means was 92.61%.

Figure 8 separates the class-level results and shows the three runs per condition. Across condition-wise means, Stone recall, mAP50–95 and IoU averaged 85.47%, 36.19% and 88.36%, compared with 98.16%, 80.71% and 96.86% for Tree. The condition-wise mean mAP50–95 ranged from 27.29% to 46.11% for Stone and 77.76% to 83.41% for Tree. Stone therefore had lower recall and mask quality, particularly under stricter IoU thresholds. Small size, irregular shape and local occlusion may contribute to this difficulty, but their effects were not isolated experimentally.

**Figure 9:**
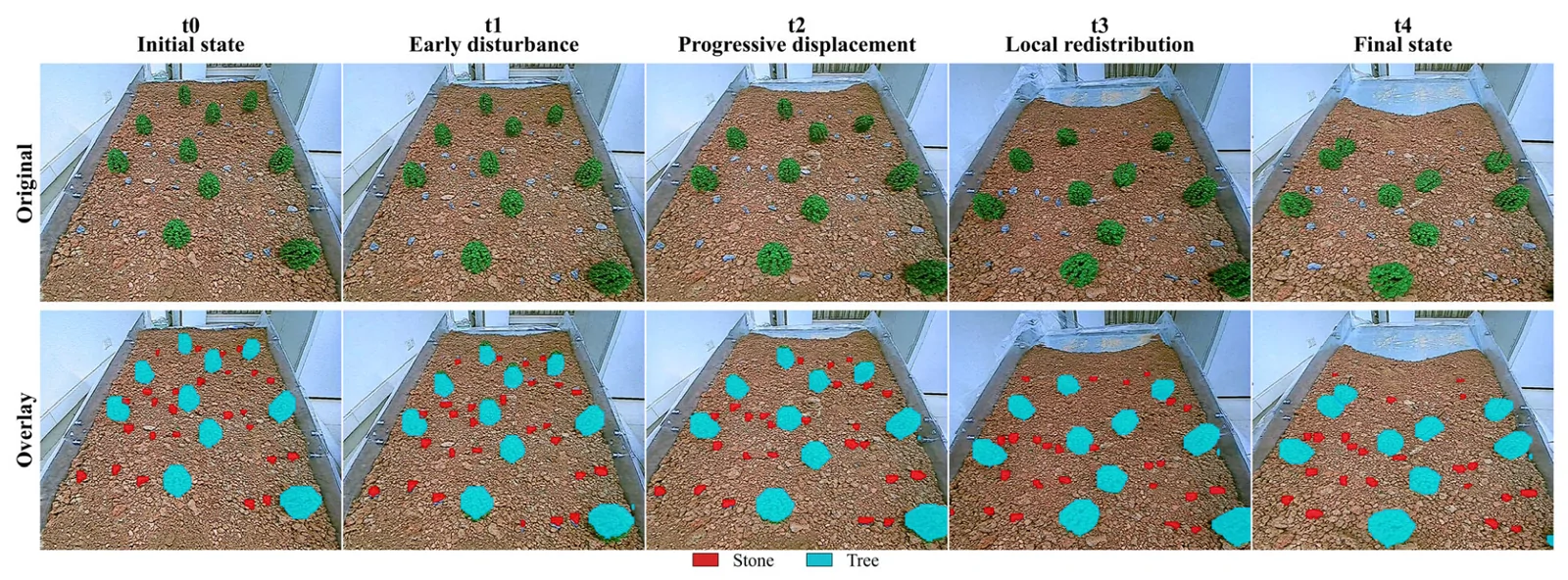
Stone and Tree instance segmentation results at selected time points under working condition C1.

Figure 9 shows C1 segmentation outputs at five selected time points (t0–t4). Original images appear above predicted masks, with Stone in red and Tree in bright cyan blue. The overlays retain the visible arrangement of clustered Tree and small Stone instances as disturbance changes their distribution and exposure. These examples show the masks supplied to subsequent tracking and spatial visualization.

#### 4.3.2. Temporal Tracking and Motion State Quantification of STONE and Tree Instances

Table 4 presents standard multi-object tracking metrics across the nine working conditions. HOTA and IDF1 summarize tracking and identity consistency, AssPr and AssRe describe association quality, and the normalized IDSW and Frag rates quantify identity switch and fragmentation events relative to target exposure; the final row gives the pooled C1–C9 results.

Across the three replicate-level pooled C1–C9 results, HOTA and IDF1 were 89.07 ± 0.17% and 92.50 ± 0.27%, respectively. AssPr was 97.75 ± 0.19%, whereas AssRe was 90.92 ± 0.25%, indicating a larger loss in association recall than in association precision. The normalized IDSW and Frag rates were 1.69 ± 0.03 and 2.73 ± 0.06 events per 1000 GT detections, respectively. These exposure-normalized rates reduce the direct influence of differences in sequence length and target exposure on cross-condition comparison.

Among the condition-wise mean scores, C7 had the lowest HOTA, IDF1 and AssRe, at 81.63%, 83.70% and 81.80%, respectively. In the normalized event rate comparison, C8 had the highest IDSW rate, at 5.51 ± 0.20 events per 1000 GT detections, whereas C1 had the highest Frag rate, at 5.43 ± 0.27 events per 1000 GT detections. C2 had no IDSW events across the three runs and the lowest Frag rate, at 0.30 ± 0.01 events per 1000 GT detections.

Thus, the conditions with the most pronounced association and identity weaknesses differ according to the type of error; the high aggregate AssPr does not remove the recall loss or the observed identity switches and fragmentation.

**Figure 10:**
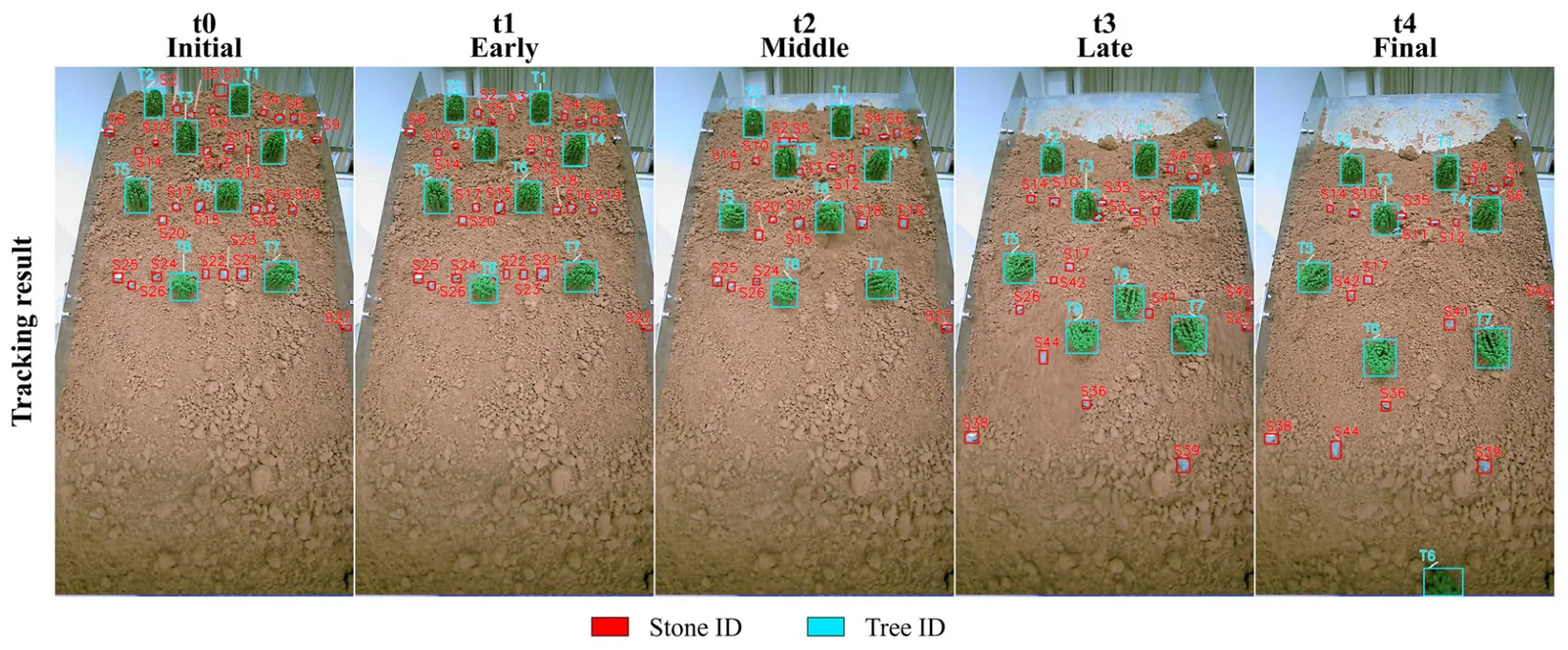
Stone and Tree identity preservation results at selected time points under working condition C7.

Figure 10 illustrates identity preservation at five representative time points in C7, the condition with the lowest HOTA, IDF1 and AssRe. Tree instances generally retained stable identity labels across the displayed time points. In this example, some Stone tracks showed short-term loss and neighboring-instance interference in locally dense regions. These observations illustrate the association difficulties of small Stone instances during occlusion and adjacent motion and complement the quantitative results.

**Figure 11:**
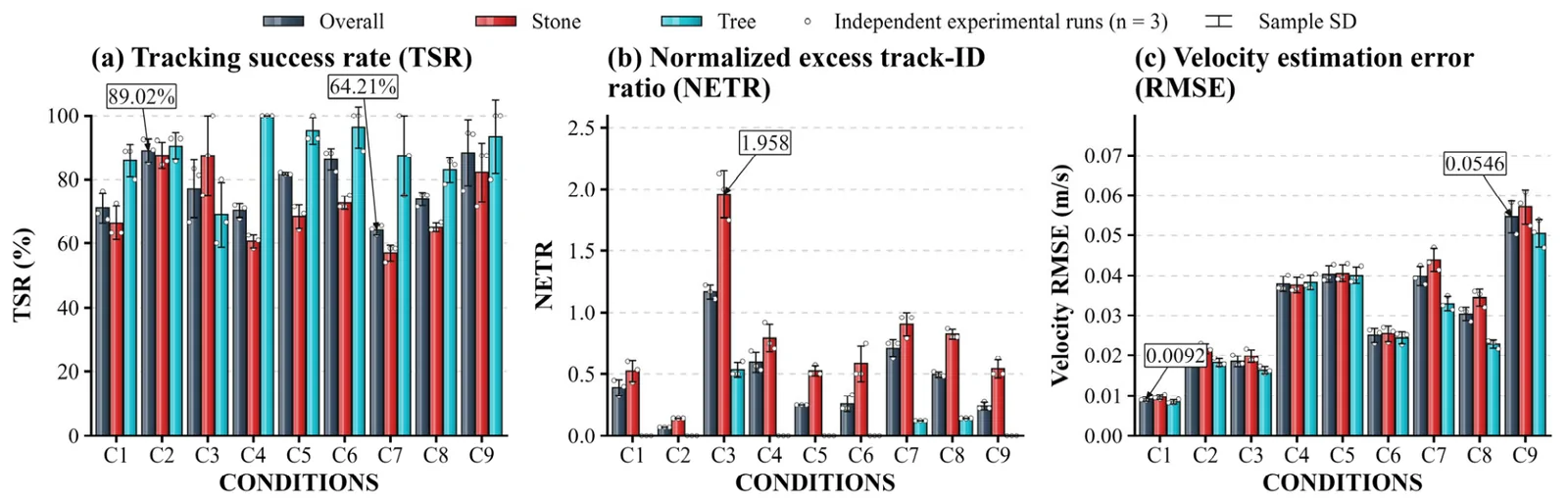
Tracking and velocity estimation results for Stone and Tree under nine working conditions: (**a**) tracking success rate (TSR); (**b**) normalized excess track-ID ratio (NETR); (**c**) velocity estimation RMSE (three independent experimental runs per condition).

Figure 11 complements the standard MOT evaluation with the application-specific TSR and NETR measures and the velocity estimation error. In Figure 11a, condition-wise mean Overall TSR ranged from 64.21% in C7 to 89.02% in C2. Across the three replicate-level pooled results, TSR was 76.45 ± 1.24% overall, 68.16 ± 1.30% for Stone and 88.77 ± 1.57% for Tree. Stone had a lower mean TSR than Tree in eight of the nine conditions and reached its minimum in C7 (56.94%); C3 was the exception, with mean TSR values of 87.50% for Stone and 68.89% for Tree. TSR characterizes target coverage by a consistent identity but does not resolve frame-level association errors.

In Figure 11b, condition-wise mean Overall NETR ranged from 0.0714 in C2 to 1.1667 in C3. Across the three replicate-level pooled results, NETR was 0.455 ± 0.029 overall, 0.708 ± 0.046 for Stone and 0.082 ± 0.010 for Tree. Stone had a higher mean NETR than Tree in all nine conditions, reaching 1.9583 in C3. The relatively high Stone TSR in C3 therefore coexisted with an excess of predicted track IDs. In that condition, Stone had an IDSW rate of 0 and a Frag rate of 1.43 ± 0.29 events per 1000 GT detections, illustrating that NETR and normalized association error rates describe related but distinct tracking behavior.

Figure 11c shows that the mean Overall velocity estimation RMSE ranged from 0.0092 m/s in C1 to 0.0546 m/s in C9. The nine condition-wise means averaged 0.0323 m/s for Stone and 0.0280 m/s for Tree. Although C9 had a higher Overall TSR and lower NETR than C1, its velocity RMSE was also higher, indicating that target coverage and excess track-ID generation alone do not determine velocity estimation accuracy. The three independently acquired run-level values and sample SD in Figure 11c show variation within each condition. Taken together, the standard MOT metrics characterize identity and association reliability, TSR and NETR describe target-level tracking continuity and excess track-ID generation, and velocity RMSE quantifies motion estimation error over valid identity-consistent trajectory segments.

#### 4.3.3. Slope Geometry Estimation and 3D Reconstruction Performance

**Figure 12:**
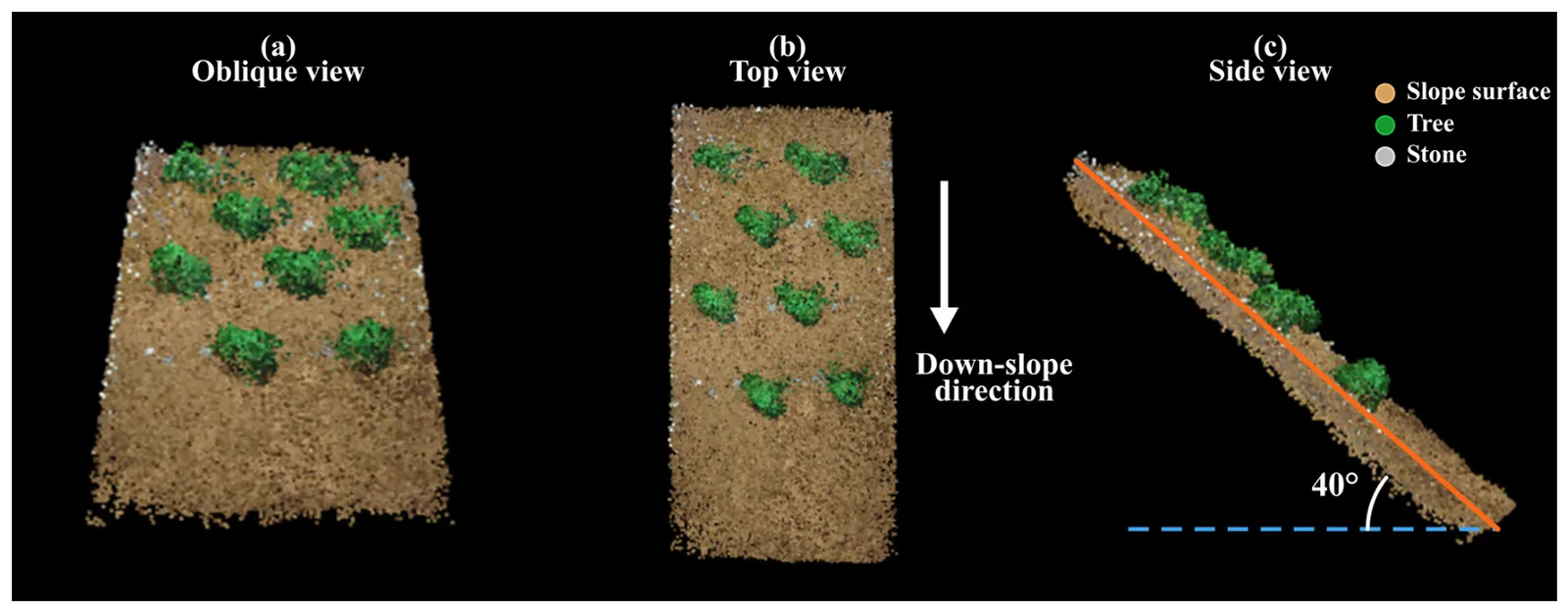
Three-view visualization of the VGGT-reconstructed full-scene point cloud under working condition C7.

Within the slope geometry estimation and 3D reconstruction module, the reconstructed slope provides the spatial context needed to interpret Stone and Tree instance locations and motion. We therefore used the full-scene point clouds generated by VGGT to express the three-dimensional spatial relationships between the dynamic slope and the Stone and Tree instances on it. Figure 12 shows oblique, top, and side views of the full-scene point cloud in working condition C7. The reconstructed point cloud visually preserved the main slope structure and the spatial arrangement of Stone and Tree instances. The slope reference angle and downslope direction annotated in the side view provide a spatial basis for later slope estimation error analysis.

**Figure 13:**
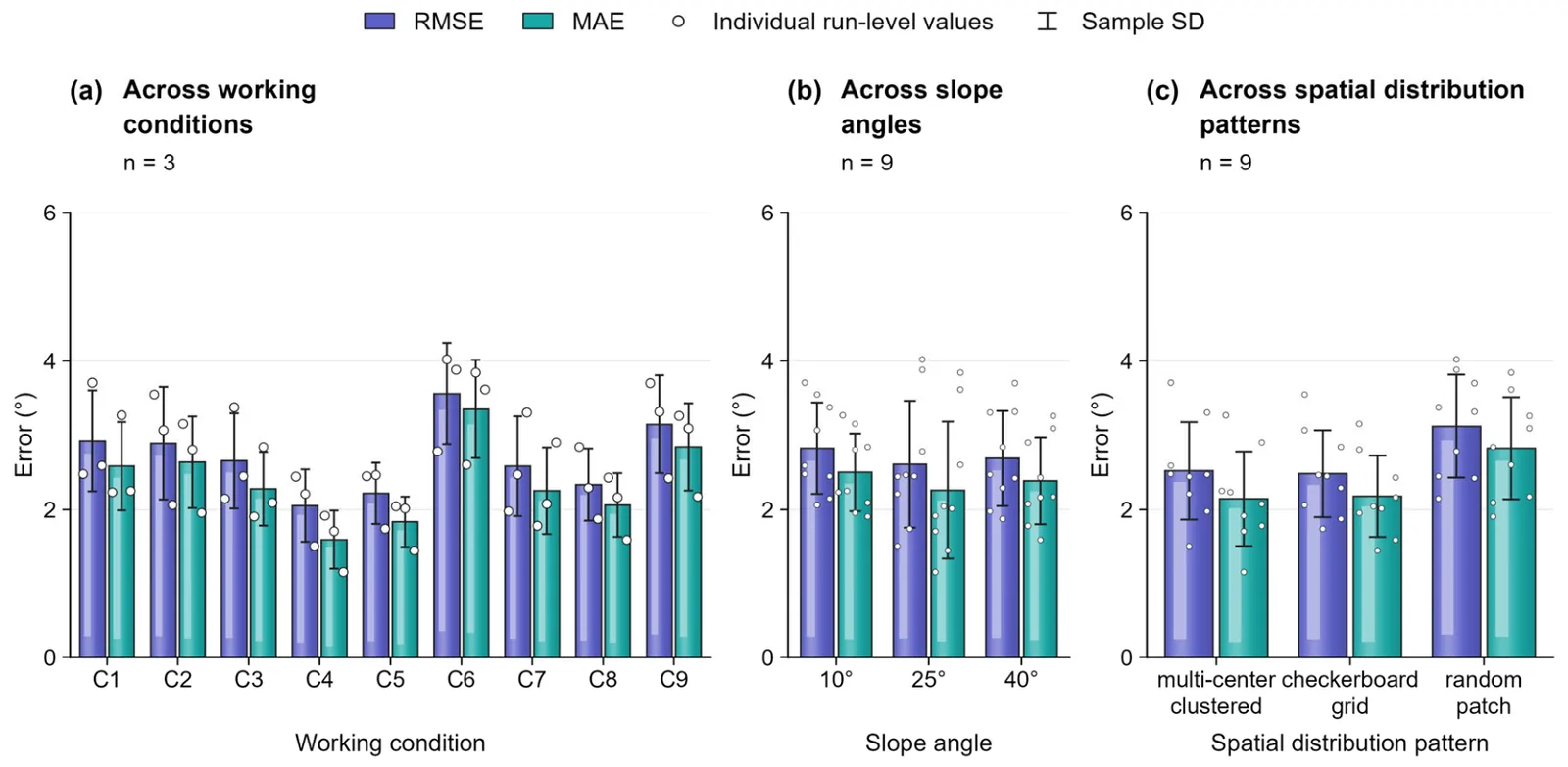
Slope estimation RMSE and MAE: (**a**) condition-wise means ± sample SD across three independent recordings per working condition; (**b**,**c**) descriptive grouped means ± sample SD by slope angle and spatial pattern labels, each based on nine run-level values from three working conditions. Open circles show individual run-level values.

Table 5 and Figure 13a summarize slope estimation errors across three independent recordings per working condition. The mean RMSE ranged from 2.0528° (C4) to 3.5596° (C6), and mean MAE from 1.5924° (C4) to 3.3529° (C6). Table 5 reports the mean ± sample SD, and Figure 13a shows the three individual results. Comparisons between working conditions were descriptive, without formal pairwise tests.

Figure 13b,c group the run-level slope errors by the slope angle and spatial pattern labels used in the nine tested configurations. Each displayed group combines three working conditions (nine runs), so its mean and sample SD summarize observed variation across these configurations. The condition-wise means and variation across repeated acquisitions are shown in Table 5 and Figure 13a.

Table 6 summarizes the pooled dimensional and slope plane positional errors of the metric-scale VGGT instance point clouds across C1–C9, using the three replicate-level pooled results.

Across the five evaluated Stone and Tree dimensions, pooled MRE values ranged from 7.092% to 7.632%. The slope plane positional RMSE was 3.247 ± 0.778 mm for Stone and 6.066 ± 1.183 mm for Tree bases. The larger absolute crown width error accompanied the larger physical scale of Tree instances, while the relative dimensional errors were similar across the evaluated dimensions. Together with the slope angle results, these measurements quantify reconstructed geometry at both scene and object levels.

Tang et al. [34] used explicit four-camera calibration and laser rangefinder reference measurements, reporting average MAE, MRE and RMSE values of 1.11 mm, 0.52% and 1.84 mm, respectively, after deep-learning-based point cloud correction. Chen et al. [35] reported a mean absolute error of 1.06 mm and a mean relative error of 0.52% for calibrated multi-camera reconstruction with adaptive point cloud correction. The present VGGT evaluation combines scene-level slope angle error with object-level dimensional and slope plane positional errors derived from physical reference measurements. Because the studies use different geometric targets and reference definitions, the comparison focuses on calibration strategy and validation scope, while each numerical error is interpreted in its task-specific unit.

Taken together, the module-wise results show variation in performance across the nine working conditions. Stone exhibited lower fine-grained segmentation performance and tracking continuity than Tree, while slope estimation errors varied across conditions. The individual experimental results and sample SDs characterize the repeatability of the framework under the tested laboratory conditions. Further work should focus on segmentation and tracking of small, irregular Stone instances and on evaluating geometry recovery across a wider range of scene configurations.

### 4.4. Integrated Visualization and Scene Interpretation

Following the module-level evaluation, the integrated outputs were examined to show instance location, motion intensity and spatial concentration within the reconstructed slope geometry. C1 (10°, multi-center clustered), C6 (25°, random patch) and C8 (40°, checkerboard grid) were selected for comparison. These configurations cover all three slope angles and spatial distribution patterns at the same vibration amplitude of 0.1 g.

Disaster response studies emphasize that operational information products should be understandable, spatially and temporally specific, and responsive to end-user needs [53,54,55,56,57]. Situation awareness and shared situational understanding are also important foundations for operational interpretation and decision-making [58,59]. These considerations motivate the visualization approach, while the present assessment examines the displayed scene changes qualitatively.

**Figure 14:**
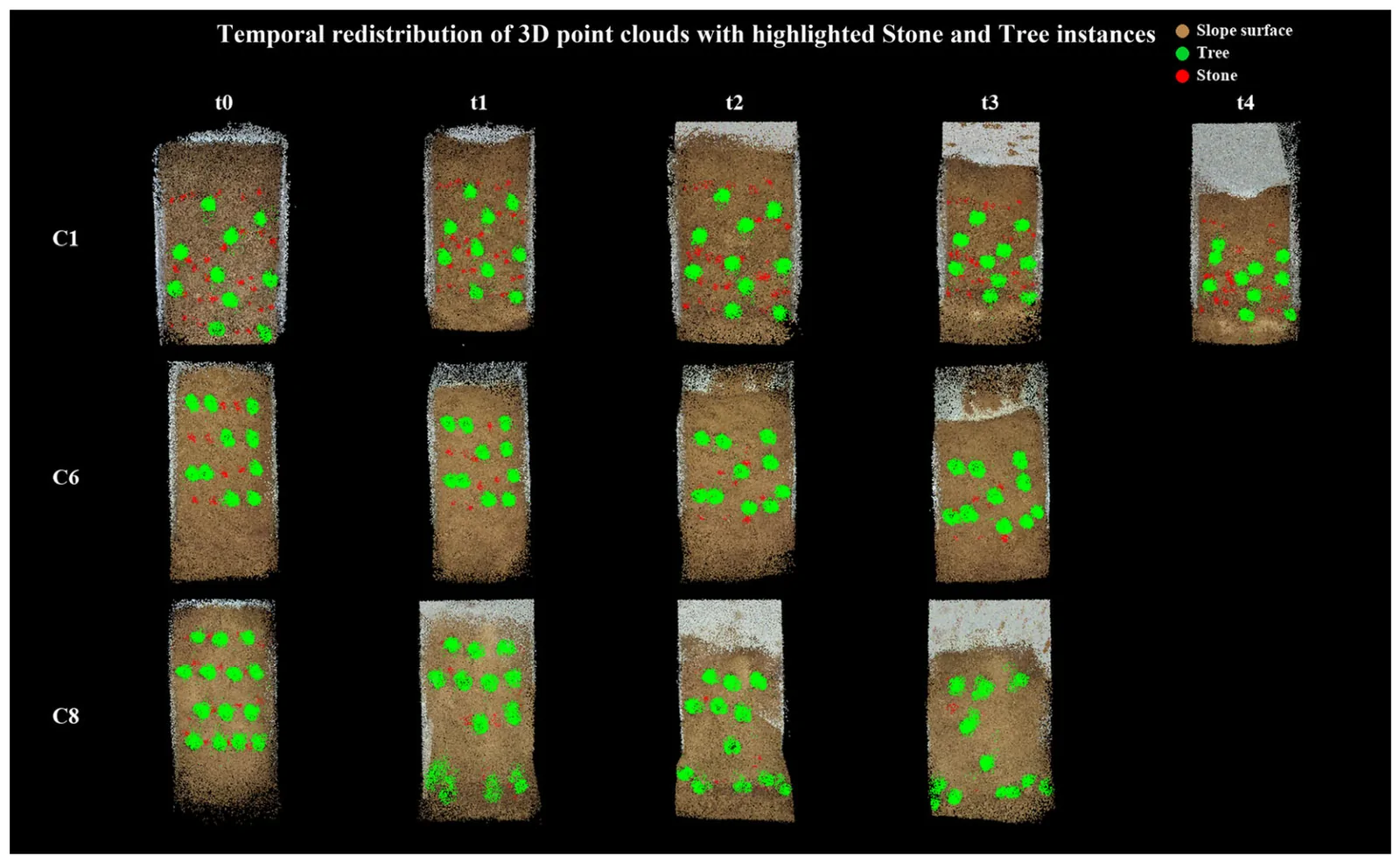
3D point clouds with highlighted Stone and Tree instances under C1, C6 and C8 at selected time points.

Figure 14 presents 3D point clouds with highlighted Stone and Tree instances at selected time points, allowing their spatial distribution to be interpreted within the reconstructed slope geometry. In C1, the instances are broadly distributed at t0–t2 and become more concentrated in the lower half of the slope at t3–t4. In C6, the distribution becomes increasingly asymmetric from t2 to t3, with more instances appearing in the lower and lateral parts of the reconstructed face. The change is most evident in C8, where the initially regular arrangement at t0 becomes progressively less uniform and several instances are located near the lower margin at later selected time points. For Stone instances, downslope redistribution and lower-slope accumulation are also spatial characteristics considered in rockfall runout analysis [60]. These point cloud visualizations therefore show how the spatial distribution of Stone and Tree instances differs across the selected observations and slope conditions.

**Figure 15:**
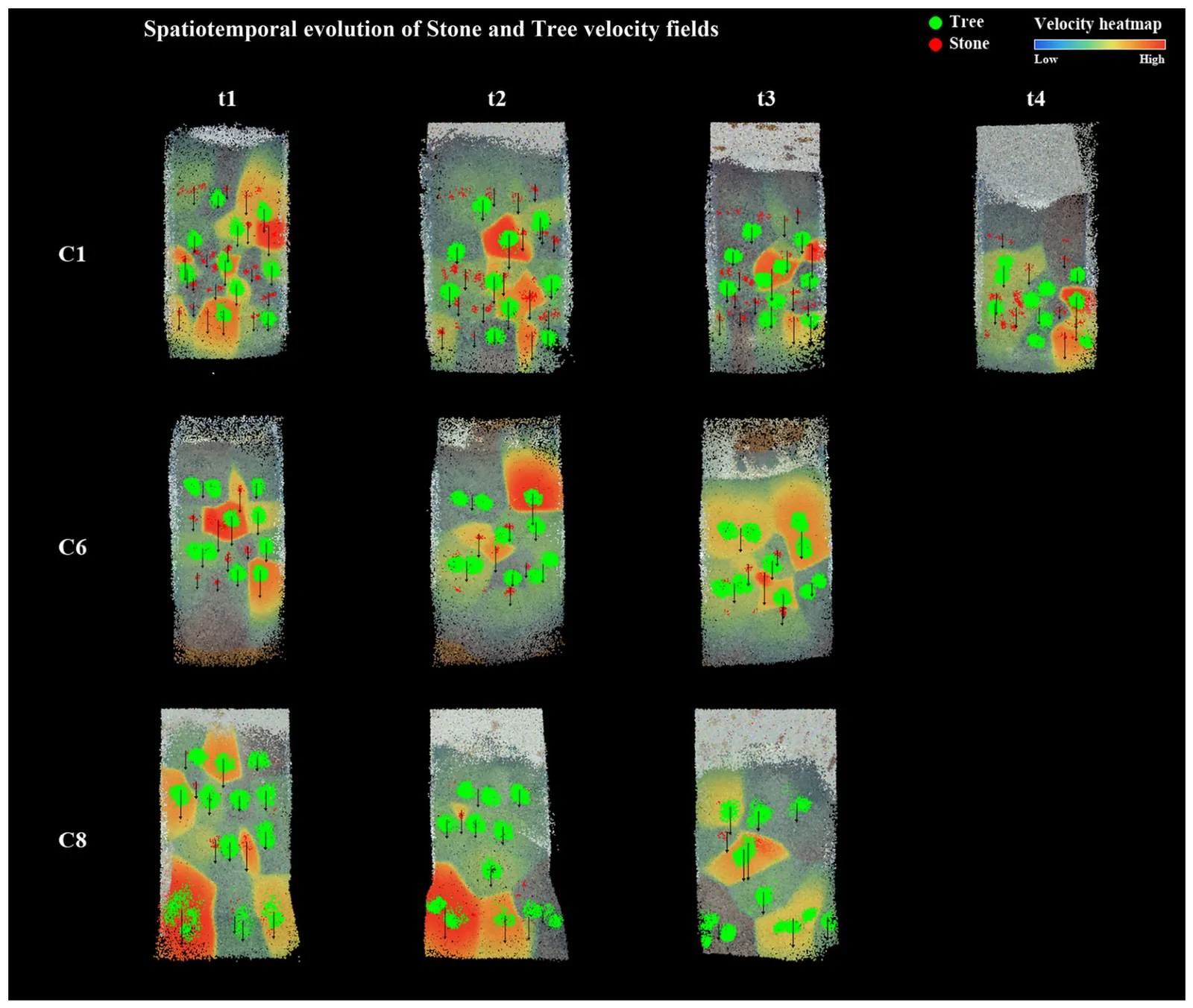
Velocity heatmaps with motion vectors for Stone and Tree instances under C1, C6 and C8 at selected time points. Arrow direction indicates movement direction, and arrow length indicates relative movement magnitude.

Figure 15 visualizes the spatial distribution of Stone and Tree motion intensity using velocity heatmaps with motion vectors under C1, C6 and C8. In C1, several moderate-to-high velocity regions are visible, with a pronounced hotspot near the central part of the slope and a later high-response region toward the lower-right area. In C6, elevated velocity is concentrated mainly in the upper-right and middle parts of the slope, showing a spatially non-uniform motion response. C8 exhibits a pronounced high-velocity response near the lower part of the slope, especially in the earlier selected frames, while the later frame shows a more spatially restricted response. The heatmap colors identify the locations of relatively high and low motion intensity, whereas the overlaid vectors provide complementary information on the direction and relative magnitude of Stone and Tree movement. Comparison across the selected time points illustrates how the spatial velocity distributions changed during disturbance.

**Figure 16:**
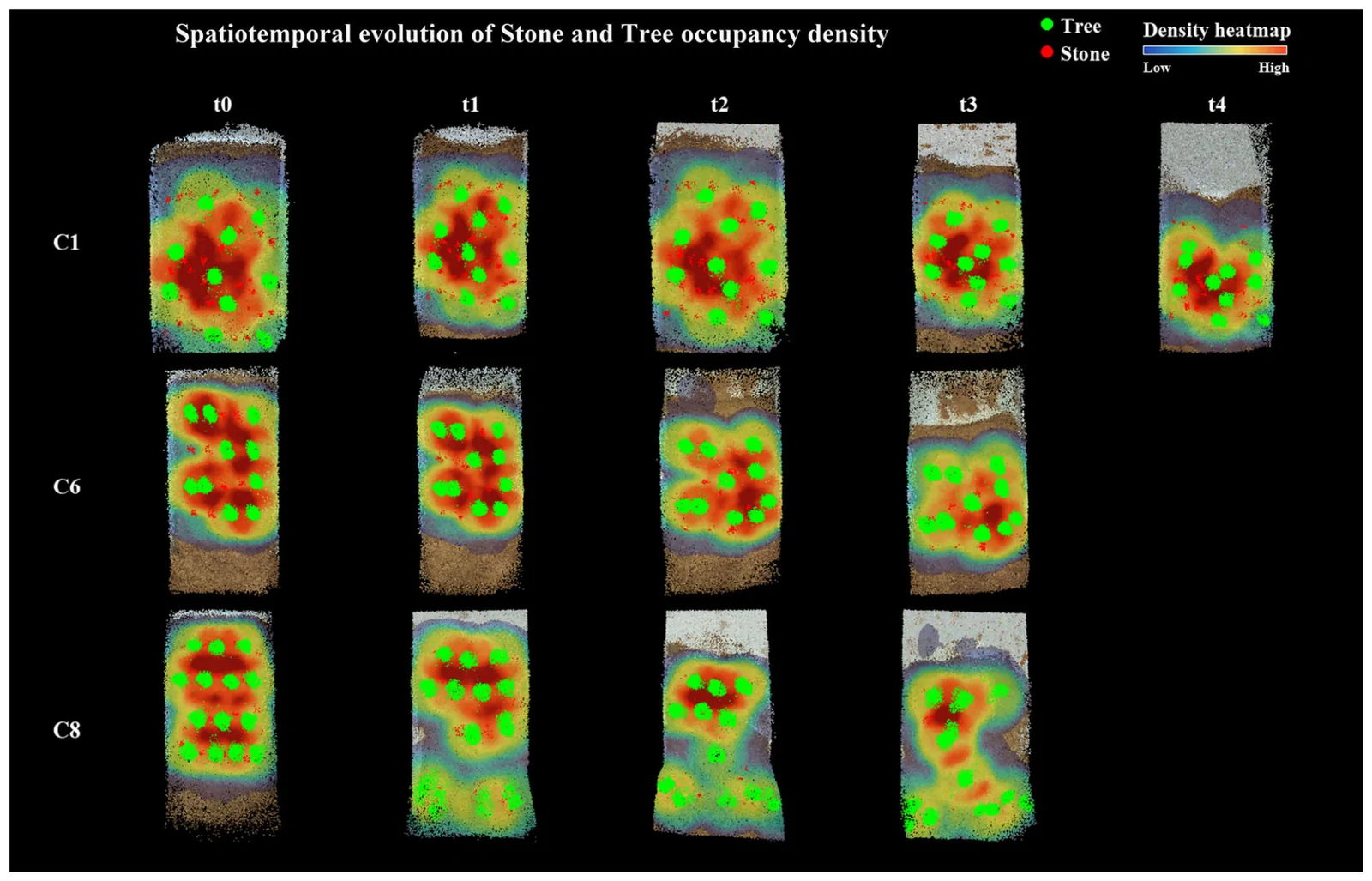
Occupancy density heatmaps for Stone and Tree instances under C1, C6 and C8 at selected time points.

Figure 16 visualizes the spatial concentration of Stone and Tree instances using occupancy density heatmaps under C1, C6 and C8. In C1, a central high-density region is evident across the selected observations and becomes more compact toward the lower-central part of the slope in the final frame. In C6, the density field changes from several distributed hotspots to a more localized concentration toward the lower-right part of the slope. In C8, the initially broad and relatively regular density pattern becomes more spatially concentrated, with an elongated central-to-lower high-density region visible in the later selected frame. The density heatmaps therefore identify where Stone and Tree instances are locally concentrated. When interpreted together with the velocity heatmaps in Figure 15, the two representations provide complementary spatial information on instance concentration and elevated motion.

Related studies help place these integrated outputs in the context of existing visual monitoring methods. Peng et al. [61] developed an embedded YOLOv8 system for road rockfall detection. The present model provides Stone and Tree masks for subsequent instance tracking and motion quantification. Table 3 compares baseline segmentation architectures and YOLOv8n-Seg optimization variants on the same model development validation subset. Relative to unscaled YOLOv8n-Seg, SSS + MGD reduced the parameter count from 3.26 to 1.03 M and Raspberry Pi 4B inference time by 59.6%. Mask mAP50–95 decreased by 0.97 percentage points, illustrating the accuracy cost of reducing computational demand.

Khan et al. [62] used time-lapse imagery and particle image velocimetry to monitor slope movement. Muhammad et al. [63] quantified displacement using optical flow and compared field estimates with interferometric synthetic aperture radar (InSAR) measurements. Brezzi et al. [64] used photogrammetry with calibrated cameras to recover spatially distributed landslide displacement. These studies demonstrate field applications of image-based motion monitoring. The present framework links Stone and Tree instances, their track identities and motion attributes to reconstructed slope geometry through pixel-to-point correspondence. This link allows instance motion to be interpreted by class and position relative to the slope surface.

Table 4 quantifies the reliability of track identities and instance associations. Figure 11c reports velocity estimation RMSE using ground-truth velocities calculated from manually tracked metric trajectories. Velocity RMSE covers only continuous, identity-consistent matched trajectory segments and does not characterize errors during missed detections or track interruptions. Table 5 and Table 6 additionally quantify scene- and object-level geometric accuracy against independent physical references. Differences in measurement targets, sampling intervals and reference data preclude direct numerical ranking against the field studies. Lower mask quality and tracking continuity for Stone remain limitations of the framework (Figure 8 and Figure 11).

The results establish module-level and integrated scene-sensing feasibility under controlled laboratory conditions. Their reliability during platform operation and in field slope environments remains to be evaluated. The test conditions did not cover the full range of spatial scales, material heterogeneity, illumination, weather, occlusion, camera motion and vibration encountered in post-earthquake or rainfall-induced landslide environments.

The stationary experiments also did not quantify excavator platform motion, platform-induced camera motion, synchronization errors under movement or end-to-end multi-view latency. The Raspberry Pi 4B ONNX times in Table 3 measure single-image model inference and do not establish sustained throughput after installation on a mobile platform. Artificial Stone and Tree instances served as controllable visual entities, rather than validated physical proxies for landslide precursors. The evidence therefore does not establish an operational landslide warning system or a fully deployed mobile excavation perception system. A staged validation pathway is required to test whether the laboratory outputs remain reliable during platform operation.

The first stage would mount synchronized, overlapping RGB cameras on a mobile excavation platform and check camera calibration, frame synchronization and slope plane referencing as the boom pose and viewing geometry change. A pilot should measure the full capture-to-display latency and sustained throughput of segmentation, ByteTrack, VGGT and visualization on the intended hardware.

Subsequent field observations should compare image-derived trajectories and slope angle estimates with independently surveyed movement, GNSS and inclinometer measurements under varying illumination, rainfall or fog, vegetation occlusion and movement speeds. Multi-site validation should then assess reliability across different slope materials, environmental conditions and camera configurations.

Candidate event detection and alert thresholds would then require site-specific evaluation of sensitivity and false alarms against observed slope movement before any integration with other monitoring sensors or an operational warning system.

## 5. Conclusions

This study evaluated a multi-view RGB framework that links instance perception and motion quantification to reconstructed slope geometry. Optimized YOLOv8n-Seg, ByteTrack and VGGT were assessed using model development data and independently collected recordings from nine laboratory conditions. The main conclusions are as follows.

(1) Static structural scaling reduced the parameter count from 3.26 M to 1.03 M and GFLOPs from 6.06 to 2.04. On the model development validation subset, SSS + MGD achieved 54.62% Mask mAP50–95, 0.97 percentage points below unscaled YOLOv8n-Seg. Inference required 4.28 ms/image on the RTX 5060 Ti and 258.70 ms/image on the Raspberry Pi 4B using ONNX. These measurements quantify the model’s accuracy–complexity trade-off.
(2) On the independent L9 recordings, the nine condition-wise means averaged 93.20% for overall mAP50 and 58.45% for mAP50–95. Condition-wise mean TSR ranged from 64.21% to 89.02%. Across the three replicate-level pooled C1–C9 results, HOTA and IDF1 were 89.07 ± 0.17% and 92.50 ± 0.27%, respectively. The mean velocity estimation RMSE ranged from 0.0092 to 0.0546 m/s. Stone had lower mean mask quality and tracking continuity than Tree. Three independently prepared recordings per condition provided run-level estimates of variability, summarized as the mean ± sample SD.
(3) Mean slope estimation RMSE ranged from 2.0528° to 3.5596° across working conditions. Against independent physical reference measurements, the metric-scale VGGT instance point clouds yielded pooled dimensional MRE values of 7.092–7.632%. These measurements covered Stone main-axis length and orthogonal width, and Tree main and orthogonal crown widths and crown height. Slope plane position RMSEs were 3.247 ± 0.778 mm for Stone centers and 6.066 ± 1.183 mm for Tree bases. The pixel-to-point correspondence from synchronized RGB frames linked instance labels to reconstructed geometry. Highlighted point clouds, velocity heatmaps with motion vectors and occupancy density heatmaps jointly represented instance location, motion intensity and spatial concentration.

The results establish laboratory-scale feasibility for integrated scene interpretation. Future work should evaluate platform-mounted sensing, moving-camera performance, end-to-end latency and field reliability under representative slope conditions.

## Figures and Tables

**Table 1:** Masked feature distillation settings and loss normalization.

Item	Implementation Used in This Experiment
Teacher and student	Baseline YOLOv8n-Seg checkpoint (frozen) and structurally scaled checkpoint; teacher.eval(), requires_grad = False and no_grad() forward pass.
Distilled outputs	model.22.cv2.{0,1,2} and model.22.cv3.{0,1,2}: box regression and classification outputs at 80 × 80, 40 × 40 and 20 × 20 (640 × 640 input).
Feature matching	Student spatial size aligned by nearest-neighbor interpolation if needed; channel-mismatched pairs skipped, without projection. K counts valid output pairs.
Spatial mask	Per output pair and forward pass: independent U ~ Uniform(0,1), shape B × 1 × H × W; M = 1[U > 0.65] broadcast over channels (about 65% masked).
Loss normalization	Per pair: mean((M ⊙ student − teacher)^2^) over all B × C × H × W elements; mean over K valid pairs, then α = 0.1. Total = task + β × masked loss, β = 0.1; task loss is scaled by batch size.
Training and seed	150 epochs; batch 16; AdamW; lr0 = 1 × 10^−4^, cosine decay (final factor 0.1), 3 warmup epochs; seed 42; deterministic = True; AMP = False; Mosaic = 0.2, off in last 20 epochs.

**Table 2:** Experimental working conditions.

Working Condition	Spatial Distribution Pattern (D)	Slope Angle (G)	Vibration Amplitude (V)	Independent Runs (*n*)	VGGT Images by Run (1/2/3)	VGGT Images, Total
C1	Multi-center clustered	10°	0.1 g	3	271/304/305	880
C2	Checkerboard grid	10°	0.3 g	3	208/251/274	733
C3	Random patch	10°	0.5 g	3	112/189/236	537
C4	Multi-center clustered	25°	0.3 g	3	126/171/205	502
C5	Checkerboard grid	25°	0.5 g	3	143/207/236	586
C6	Random patch	25°	0.1 g	3	198/224/261	683
C7	Multi-center clustered	40°	0.5 g	3	317/186/230	733
C8	Checkerboard grid	40°	0.1 g	3	157/268/281	706
C9	Random patch	40°	0.3 g	3	132/176/242	550

Abbreviation: VGGT, Visual Geometry Grounded Transformer.

**Table 3:** Network architectures and training variants of the instance segmentation model.

Network Architecture	SSS	MGD	Weight Initialization	Epochs	Box mAP50–95 (%)	Mask mAP50–95 (%)	Parameters (M)	GFLOPs	Model Size (MB)	Inference Time (ms/Image)	
										RTX 5060 Ti	Raspberry Pi 4B (ONNX)
YOLOv8n-Seg			Random initialization	150	87.5	55.59	3.26	6.06	6.46	4.71	639.90
YOLO11n-Seg			Random initialization	150	87.1	55.32	3.39	6.18	6.85	5.56	646.54
YOLO26n-Seg			Random initialization	150	86.1	55.38	3.47	6.44	7.02	6.24	664.41
YOLOv8n-Seg	✓		Random initialization	150	77.45	54.0	1.03	2.04	2.2	4.84	266.76
YOLOv8n-Seg	✓		Trained YOLOv8n-Seg baseline	150	80.1	54.42	1.03	2.04	2.2	4.35	260.97
YOLOv8n-Seg	✓	✓	Trained YOLOv8n-Seg baseline	150	85.3	54.62	1.03	2.04	2.2	4.28	258.70

Note. Box and Mask mAP50–95 are percentages. Raspberry Pi 4B results are Open Neural Network Exchange (ONNX) measurements; the GFLOPs column denotes billions of floating-point operations. SSS, static structural scaling; MGD, Masked Generative Distillation; the MGD column denotes the MGD-inspired masked feature distillation used here. All models were trained for 150 epochs. For each model, the checkpoint with the highest validation fitness, defined as the sum of Box and Mask mAP50–95, was selected for evaluation. ✓ indicates that the corresponding method or setting was used.

**Table 4:** Standard multi-object tracking metrics and normalized IDSW/Frag event rates by working condition and pooled across C1–C9 (mean ± sample SD across three independent acquisitions).

Condition	HOTA (%)	IDF1 (%)	AssPr (%)	AssRe (%)	IDSW (per 1000 GT Detections)	Frag (per 1000 GT Detections)
C1	86.55 ± 0.62	91.26 ± 1.17	97.43 ± 0.84	89.69 ± 1.04	2.34 ± 0.05	5.43 ± 0.27
C2	95.00 ± 1.17	98.04 ± 0.67	98.67 ± 1.01	96.88 ± 1.10	0.00 ± 0.00	0.30 ± 0.01
C3	89.03 ± 3.01	92.71 ± 1.48	97.02 ± 0.78	89.61 ± 2.41	0.67 ± 0.07	0.81 ± 0.13
C4	86.13 ± 1.31	88.14 ± 0.55	97.52 ± 0.32	87.21 ± 0.51	2.49 ± 0.10	4.32 ± 0.12
C5	89.38 ± 0.88	93.71 ± 0.39	96.63 ± 1.20	92.07 ± 1.31	0.70 ± 0.02	1.10 ± 0.11
C6	91.27 ± 1.67	94.43 ± 0.66	97.31 ± 1.69	91.90 ± 1.66	2.27 ± 0.07	1.09 ± 0.07
C7	81.63 ± 2.13	83.70 ± 0.80	96.91 ± 1.53	81.80 ± 2.39	1.93 ± 0.01	4.27 ± 0.40
C8	85.50 ± 1.26	89.04 ± 0.59	97.23 ± 1.68	86.29 ± 0.89	5.51 ± 0.20	5.32 ± 0.20
C9	93.04 ± 3.89	96.52 ± 1.76	97.91 ± 0.99	93.85 ± 3.44	0.36 ± 0.01	1.50 ± 0.14
**Pooled C1–C9**	**89.07 ± 0.17**	**92.50 ± 0.27**	**97.75 ± 0.19**	**90.92 ± 0.25**	**1.69 ± 0.03**	**2.73 ± 0.06**

HOTA, IDF1, AssPr and AssRe are percentages. IDSW and Frag are events per 1000 ground-truth (GT) detections, calculated for each recording as the event count divided by the GT detection count and multiplied by 1000. Each condition was independently acquired three times (*n* = 3); the table reports the mean ± sample SD of the three run-level values. For each of R1–R3, pooled HOTA, IDF1, AssPr and AssRe were calculated across the nine corresponding C1–C9 recordings using TrackEval’s metric-specific aggregation. Pooled IDSW and Frag rates used the summed event and GT detection counts from these nine recordings. The pooled mean and SD summarize the three replicate-level pooled results. Bold type identifies the pooled C1–C9 summary.

**Table 5:** Slope estimation error across three independent runs per working condition.

Condition	RMSE (Mean ± SD, °)	MAE (Mean ± SD, °)
C1	2.93 ± 0.68	2.59 ± 0.60
C2	2.89 ± 0.76	2.64 ± 0.62
C3	2.66 ± 0.64	2.28 ± 0.50
C4	2.05 ± 0.49	1.59 ± 0.39
C5	2.22 ± 0.41	1.83 ± 0.34
C6	3.56 ± 0.68	3.35 ± 0.66
C7	2.59 ± 0.67	2.25 ± 0.58
C8	2.34 ± 0.49	2.06 ± 0.43
C9	3.15 ± 0.66	2.84 ± 0.59

**Table 6:** Pooled object-level geometric accuracy across C1–C9 (mean ± sample SD, *n* = 3).

Object	Geometric Metric	Mean ± SD	Unit
Stone	Main-axis length MAE	2.217 ± 0.265	mm
Stone	Main-axis length MRE	7.092 ± 0.661	%
Stone	Orthogonal width MAE	1.269 ± 0.184	mm
Stone	Orthogonal width MRE	7.279 ± 0.975	%
Stone	Slope plane position RMSE	3.247 ± 0.778	mm
Tree	Main crown width MAE	9.075 ± 1.684	mm
Tree	Main crown width MRE	7.218 ± 0.764	%
Tree	Orthogonal crown width MAE	3.300 ± 0.090	mm
Tree	Orthogonal crown width MRE	7.477 ± 0.121	%
Tree	Crown height MAE	4.898 ± 0.548	mm
Tree	Crown height MRE	7.632 ± 0.786	%
Tree	Slope plane base position RMSE	6.066 ± 1.183	mm

Note. For each of R1–R3, measurements from the nine corresponding C1–C9 recordings were pooled, comprising 128 Stone and 96 Tree objects per pooled replicate. The mean and sample SD were calculated from the three pooled values for each metric (*n* = 3).

## Data Availability

The datasets generated and/or analyzed during the current study are available from the corresponding author upon reasonable request. The data are not publicly available due to confidentiality and contractual restrictions associated with the collaborative project.

## References

[1] Frese C., Zube A., Woock P., Emter T., Heide N.F., Albrecht A., Petereit J. (2022). An Autonomous Crawler Excavator for Hazardous Environments. at-Automatisierungstechnik.

[2] Rasul A., Seo J., Oh K., Khajepour A., Reginald N. Predicted Safety Algorithms for Autonomous Excavators Using a 3D LiDAR Sensor. Proceedings of the 2020 IEEE International Systems Conference (SysCon).

[3] Rasul A., Seo J., Khajepour A. (2021). Development of Sensing Algorithms for Object Tracking and Predictive Safety Evaluation of Autonomous Excavators. Appl. Sci..

[4] Fu S., Cai Q., Li Z., Wang W., Lin T., Chen Q., Yao Z. (2026). Semantic Segmentation and Effect Optimization of 3D Point Cloud Based on 2D Semantic Segmentation and Clustering for Construction Machinery Unstructured Environment. Sensors.

[5] Hinze J.W., Teizer J. (2011). Visibility-related Fatalities Related to Construction Equipment. Saf. Sci..

[6] Ray S.J., Teizer J. (2013). Computing 3D blind spots of construction equipment: Implementation and evaluation of an automated measurement and visualization method utilizing range point cloud data. Autom. Constr..

[7] Ray S.J., Teizer J. (2016). Dynamic Blindspots Measurement for Construction Equipment Operators. Saf. Sci..

[8] Teizer J., Allread B.S., Mantripragada U. (2010). Automating the Blind Spot Measurement of Construction Equipment. Autom. Constr..

[9] Chigira M., Tsou C.-Y., Matsushi Y., Hiraishi N., Matsuzawa M. (2013). Topographic Precursors and Geological Structures of Deep-Seated Catastrophic Landslides Caused by Typhoon Talas. Geomorphology.

[10] Roberti G., Ward B., van Wyk de Vries B., Friele P., Perotti L., Clague J.J., Giardino M. (2017). Precursory Slope Distress Prior to the 2010 Mount Meager Landslide, British Columbia. Landslides.

[11] Xu Q., Zeng Y. (2009). Research on Acceleration Variation Characteristics of Creep Landslide and Early-Warning Prediction Indicator of Critical Sliding. Chin. J. Rock Mech. Eng..

[12] Huang R., Chen G., Tang P. (2017). Precursor information of locking segment landslides based on transient characteristics. Chin. J. Rock Mech. Eng..

[13] Intrieri E., Gigli G., Mugnai F., Fanti R., Casagli N. (2012). Design and Implementation of a Landslide Early Warning System. Eng. Geol..

[14] Atzeni C., Barla M., Pieraccini M., Antolini F. (2015). Early Warning Monitoring of Natural and Engineered Slopes with Ground-Based Synthetic-Aperture Radar. Rock Mech. Rock Eng..

[15] Huang G., Du S., Wang D. (2023). GNSS Techniques for Real-Time Monitoring of Landslides: A Review. Satell. Navig..

[16] Komac M., Holley R., Mahapatra P., van der Marel H., Bavec M. (2014). Coupling of GPS/GNSS and Radar Interferometric Data for a 3D Surface Displacement Monitoring of Landslides. Landslides.

[17] Pei H.-F., Yin J.-H., Zhu H.-H., Hong C.-Y., Jin W., Xu D.-S. (2012). Monitoring of Lateral Displacements of a Slope Using a Series of Special Fibre Bragg Grating-Based In-Place Inclinometers. Meas. Sci. Technol..

[18] Tarchi D., Casagli N., Moretti S., Leva D., Sieber A. (2003). Monitoring Landslide Displacements by Using Ground-based Synthetic Aperture Radar Interferometry: Application to the Ruinon Landslide in the Italian Alps. J. Geophys. Res. Solid Earth.

[19] Dong H., Sheng S., Xu C. (2025). A Review of Video-Based Monitoring Systems for Geohazard Early Warning. Sensors.

[20] Wang Q., Chen D., Feng W., Sun L., Yu G. (2025). Enhancing Instance Segmentation in Agriculture: An Optimized YOLOv8 Solution. Sensors.

[21] Li C., Jiang S., Cao X. (2025). Small-Target Detection Algorithm Based on STDA-YOLOv8. Sensors.

[22] Yang C., Shen Y., Wang L. (2025). EMFE-YOLO: A Lightweight Small Object Detection Model for UAVs. Sensors.

[23] Wang L., Niu H., Wang T., Wang Q., Niu Q. (2026). SPR-YOLOv8: A Real-Time Instance Segmentation and Dynamic Size Measurement System for Diamond Particles. Sensors.

[24] Sayed M., Brostow G. Improved Handling of Motion Blur in Online Object Detection. Proceedings of the IEEE/CVF Conference on Computer Vision and Pattern Recognition (CVPR).

[25] Yang X., Wang G., Hu W., Gao J., Lin S., Li L., Gao K., Wang Y. Video Tiny-Object Detection Guided by the Spatial-Temporal Motion Information. Proceedings of the IEEE/CVF Conference on Computer Vision and Pattern Recognition Workshops (CVPRW).

[26] Zhang Y., Sun P., Jiang Y., Yu D., Weng F., Yuan Z., Luo P., Liu W., Wang X. ByteTrack: Multi-Object Tracking by Associating Every Detection Box. Proceedings of the European Conference on Computer Vision (ECCV).

[27] Dendorfer P., Osep A., Milan A., Schindler K., Cremers D., Reid I., Roth S., Leal-Taixé L. (2021). MOTChallenge: A Benchmark for Single-Camera Multiple Target Tracking. Int. J. Comput. Vis..

[28] Zhang Z., Wu F., Qiu Y., Liang J., Li S., Wang L., Gall J., Chin T.J., Sato I., Chellappa R. (2023). Tracking Small and Fast Moving Objects: A Benchmark. Computer Vision—ACCV 2022.

[29] Liu W., Yao J., Jiang F., Wang M. (2025). An Improved Multi-Object Tracking Algorithm Designed for Complex Environments. Sensors.

[30] Ju C., Zhao Z., Shen C., Li X., Namiki A. (2026). MOT-Assisted Object-Level Point Cloud Extraction from Multi-View Observations. Sensors.

[31] Wang H., Zhu R., Ye Z., Li Y. (2026). Robot Object Detection and Tracking Based on Image–Point Cloud Instance Matching. Sensors.

[32] Hu K., Chen Z., Kang H., Tang Y. (2024). 3D Vision Technologies for a Self-Developed Structural External Crack Damage Recognition Robot. Autom. Constr..

[33] Duan H., Hu S., Wang F., Shimasaki K., Ishii I. (2026). Multi-Frame Temporal Integration for 3-D Shape Measurement of Freely Falling Small Objects Using a High-Speed Camera Array. Sensors.

[34] Tang Y., Chen M., Lin Y., Huang X., Huang K., He Y., Li L. (2020). Vision-Based Three-Dimensional Reconstruction and Monitoring of Large-Scale Steel Tubular Structures. Adv. Civ. Eng..

[35] Chen M., Tang Y., Zou X., Huang K., Li L., He Y. (2019). High-Accuracy Multi-Camera Reconstruction Enhanced by Adaptive Point Cloud Correction Algorithm. Opt. Lasers Eng..

[36] Chen Z., Zhang C., Tang Z., Fang K., Xu W. (2024). Three-Dimensional Reconstruction and Deformation Identification of Slope Models Based on Structured Light Method. Sensors.

[37] Luo W., Lu Z., Liao Q. (2024). LNMVSNet: A Low-Noise Multi-View Stereo Depth Inference Method for 3D Reconstruction. Sensors.

[38] Ranftl R., Lasinger K., Hafner D., Schindler K., Koltun V. (2022). Towards Robust Monocular Depth Estimation: Mixing Datasets for Zero-shot Cross-dataset Transfer. IEEE Trans. Pattern Anal. Mach. Intell..

[39] Yang L., Kang B., Huang Z., Xu X., Feng J., Zhao H. Depth Anything: Unleashing the Power of Large-Scale Unlabeled Data. Proceedings of the IEEE/CVF Conference on Computer Vision and Pattern Recognition (CVPR).

[40] Wang J., Chen M., Karaev N., Vedaldi A., Rupprecht C., Novotny D. VGGT: Visual Geometry Grounded Transformer. Proceedings of the IEEE/CVF Conference on Computer Vision and Pattern Recognition (CVPR).

[41] Yang Z., Li Z., Shao M., Shi D., Yuan Z., Yuan C. Masked Generative Distillation. Proceedings of the European Conference on Computer Vision (ECCV).

[42] Tan M., Le Q.V. EfficientNet: Rethinking model scaling for convolutional neural networks. Proceedings of the 36th International Conference on Machine Learning (ICML).

[43] Lu X., Cao Y., Chen S., Li W., Zhou X., Lu X. (2024). Distilling Object Detectors with Efficient Logit Mimicking and Mask-Guided Feature Imitation. Expert Syst. Appl..

[44] Yang G., Tang Y., Wu Z., Li J., Xu J., Wan X. DMKD: Improving Feature-Based Knowledge Distillation for Object Detection via Dual Masking Augmentation. Proceedings of the IEEE International Conference on Acoustics, Speech and Signal Processing (ICASSP).

[45] Guo G., Zhao S. (2023). 3D Multi-Object Tracking with Adaptive Cubature Kalman Filter for Autonomous Driving. IEEE Trans. Intell. Veh..

[46] Kawtikwar S., Nagi R. Multi-Target Tracking with GPU-Accelerated Data Association Engine. Proceedings of the 2023 26th International Conference on Information Fusion (FUSION).

[47] Padilla R., Passos W.L., Dias T.L.B., Netto S.L., da Silva E.A.B. (2021). A Comparative Analysis of Object Detection Metrics with a Companion Open-Source Toolkit. Electronics.

[48] Ahmadzadeh A., Kempton D.J., Chen Y., Angryk R.A. Multiscale IoU: A Metric for Evaluation of Salient Object Detection with Fine Structures. Proceedings of the IEEE International Conference on Image Processing (ICIP).

[49] Luiten J., Osep A., Dendorfer P., Torr P., Geiger A., Leal-Taixé L., Leibe B. (2020). HOTA: A Higher Order Metric for Evaluating Multi-object Tracking. Int. J. Comput. Vis..

[50] Ristani E., Solera F., Zou R., Cucchiara R., Tomasi C., Hua G., Jégou H. (2016). Performance Measures and a Data Set for Multi-Target, Multi-Camera Tracking. Computer Vision—ECCV 2016 Workshops.

[51] Bernardin K., Stiefelhagen R. (2008). Evaluating Multiple Object Tracking Performance: The CLEAR MOT Metrics. EURASIP J. Image Video Process..

[52] Zhang G., Chen Y. A Metric for Evaluating 3D Reconstruction and Mapping Performance with No Ground Truthing. Proceedings of the IEEE International Conference on Image Processing (ICIP).

[53] Perera D., Agnihotri J., Seidou O., Djalante R. (2020). Identifying societal challenges in flood early warning systems. Int. J. Disaster Risk Reduct..

[54] Neußner O. (2021). Early warning alerts for extreme natural hazard events: A review of worldwide practices. Int. J. Disaster Risk Reduct..

[55] Mehta A.M., Greer D.A., Weeks C.S., Schuster L., Murray S., Dootson P., Richards A. (2022). Mind the gap: Contrasting operational and behavior-oriented flood warnings. Int. J. Disaster Risk Reduct..

[56] Twomlow A., Grainger S., Cieslik K., Paul J.D., Buytaert W. (2022). A user-centred design framework for disaster risk visualisation. Int. J. Disaster Risk Reduct..

[57] Kox T., Kempf H., Lüder C., Hagedorn R., Gerhold L. (2018). Towards user-orientated weather warnings. Int. J. Disaster Risk Reduct..

[58] O’Brien A., Read G.J.M., Salmon P.M. (2020). Situation awareness in multi-agency emergency response: Models, methods and applications. Int. J. Disaster Risk Reduct..

[59] Laurila-Pant M., Pihlajamäki M., Lanki A., Lehikoinen A. (2023). A protocol for analysing the role of shared situational awareness and decision-making in cooperative disaster simulations. Int. J. Disaster Risk Reduct..

[60] Sarkar N., Devoto S., Vandelli V., Rossi S., Soldati M., Rizzo A. (2024). Rock-fall Runout Simulation Using a QGIS Plugin along the North-West Coast of Malta (Mediterranean Sea). Nat. Hazards.

[61] Peng P., Gao L., Li J., Zhang H. (2025). Optimized YOLOv8 framework for intelligent rockfall detection on mountain roads. Sci. Rep..

[62] Khan M.W., Dunning S., Bainbridge R., Martin J., Diaz-Moreno A., Torun H., Jin N., Woodward J., Lim M. (2021). Low-Cost Automatic Slope Monitoring Using Vector Tracking Analyses on Live-Streamed Time-Lapse Imagery. Remote Sens..

[63] Muhammad M., Williams-Jones G., Stead D., Tortini R., Falorni G., Donati D. (2022). Applications of Image-Based Computer Vision for Remote Surveillance of Slope Instability. Front. Earth Sci..

[64] Brezzi L., Gabrieli F., Vallisari D., Carraro E., Pol A., Galgaro A., Cola S. (2024). DIPHORM: An Innovative DIgital PHOtogrammetRic Monitoring Technique for Detecting Surficial Displacements of Landslides. Remote Sens..

